# Evolutionary Divergence of the Wsp Signal Transduction Systems in Beta- and Gammaproteobacteria

**DOI:** 10.1128/AEM.01306-21

**Published:** 2021-10-28

**Authors:** Collin Kessler, Eisha Mhatre, Vaughn Cooper, Wook Kim

**Affiliations:** a Department of Biological Sciences, Duquesne University, Pittsburgh, Pennsylvania, USA; b Department of Microbiology and Molecular Genetics, University of Pittsburgh, Pittsburgh, Pennsylvania, USA; Nanjing Agricultural University

**Keywords:** biofilms, phylogenetic analysis, mutational studies, *Pseudomonas*, *Burkholderia*, signal transduction

## Abstract

Bacteria rapidly adapt to their environment by integrating external stimuli through diverse signal transduction systems. Pseudomonas aeruginosa, for example, senses surface contact through the Wsp signal transduction system to trigger the production of cyclic di-GMP. Diverse mutations in *wsp* genes that manifest enhanced biofilm formation are frequently reported in clinical isolates of P. aeruginosa and in biofilm studies of Pseudomonas spp. and Burkholderia cenocepacia. In contrast to the convergent phenotypes associated with comparable *wsp* mutations, we demonstrate that the Wsp system in B. cenocepacia does not impact intracellular cyclic di-GMP levels, unlike that in Pseudomonas spp. Our current mechanistic understanding of the Wsp system is based entirely on the study of four Pseudomonas spp., and its phylogenetic distribution remains unknown. Here, we present a broad phylogenetic analysis to show that the Wsp system originated in the betaproteobacteria and then horizontally transferred to Pseudomonas spp., the sole member of the gammaproteobacteria. Alignment of 794 independent Wsp systems with reported mutations from the literature identified key amino acid residues that fall within and outside annotated functional domains. Specific residues that are highly conserved but uniquely modified in B. cenocepacia likely define mechanistic differences among Wsp systems. We also find the greatest sequence variation in the extracellular sensory domain of WspA, indicating potential adaptations to diverse external stimuli beyond surface contact sensing. This study emphasizes the need to better understand the breadth of functional diversity of the Wsp system as a major regulator of bacterial adaptation beyond B. cenocepacia and select Pseudomonas spp.

**IMPORTANCE** The Wsp signal transduction system serves as an important model system for studying how bacteria adapt to living in densely structured communities known as biofilms. Biofilms frequently cause chronic infections and environmental fouling, and they are very difficult to eradicate. In Pseudomonas aeruginosa, the Wsp system senses contact with a surface, which in turn activates specific genes that promote biofilm formation. We demonstrate that the Wsp system in Burkholderia cenocepacia regulates biofilm formation uniquely from that in Pseudomonas species. Furthermore, a broad phylogenetic analysis reveals the presence of the Wsp system in diverse bacterial species, and sequence analyses of 794 independent systems suggest that the core signaling components function similarly but with key differences that may alter what or how they sense. This study shows that Wsp systems are highly conserved and more broadly distributed than previously thought, and their unique differences likely reflect adaptations to distinct environments.

## INTRODUCTION

Biofilms are extremely recalcitrant in nature, which is driven largely by the extracellular matrix comprising diverse compounds produced by individual cells (1–8). This matrix manifests structured community growth and dynamically generates sharp chemical gradients to drive both phenotypic and genetic diversification. Exopolysaccharides (EPSs) are a major component of this matrix, and they play a critical role in cell-cell and cell surface adhesion (9–12). EPS production or export is modulated by the second messenger cyclic di-GMP (5, 13–21) in many organisms, which promotes the persistence of opportunistic pathogens like Pseudomonas aeruginosa for years in the lungs of cystic fibrosis patients and causes extensive damage (17, 22, 23). Clinical isolates of P. aeruginosa often display phenotypic heterogeneity as either smooth, mucoid, or small colony variant (SCV) phenotypes (22, 24–26). Sequence analyses of SCVs commonly identify mutations in *wsp* (wrinkly spreader phenotype) genes that increase the intracellular pool of cyclic di-GMP (27–31).

**FIG 1:**
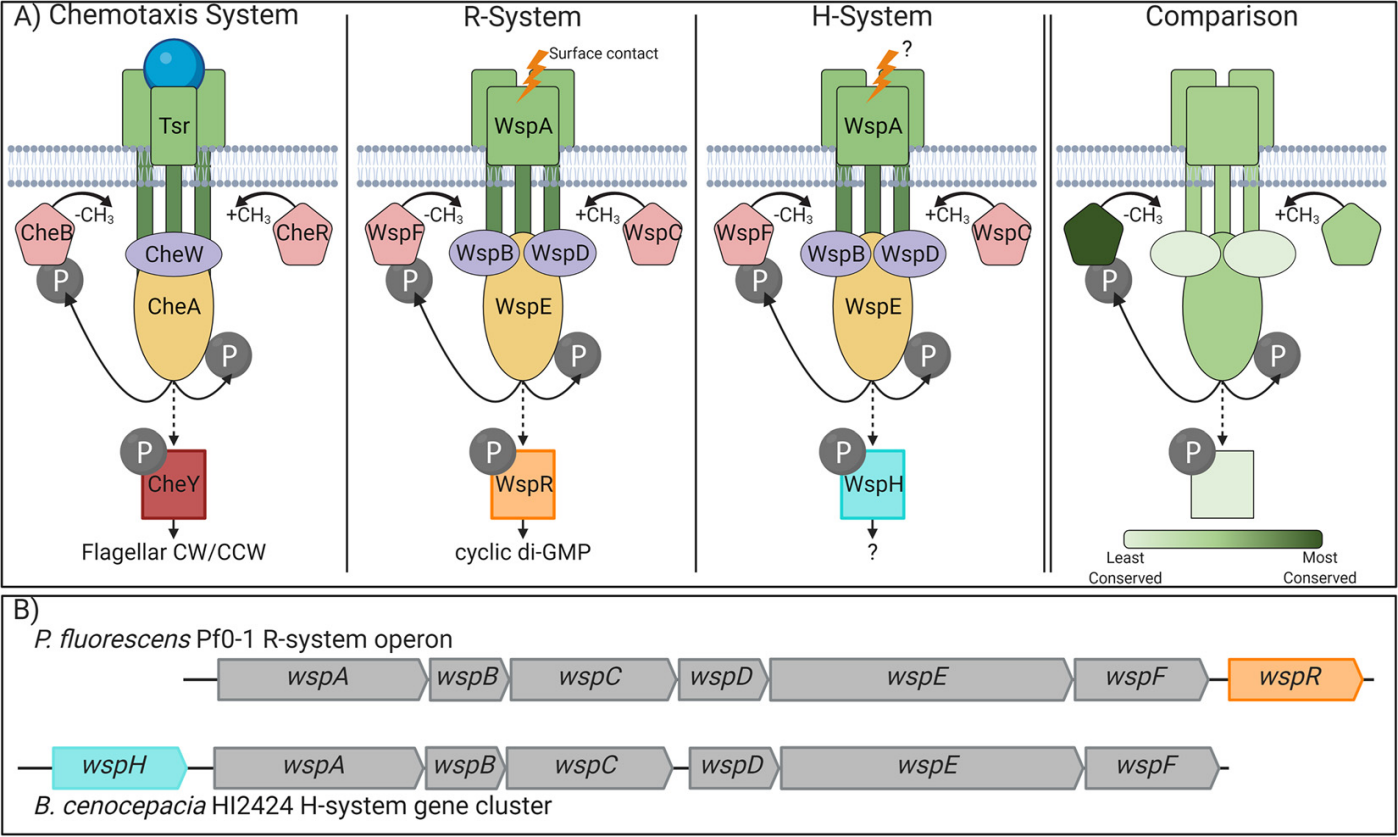
Comparison of the Wsp signal transduction system to the enteric chemotaxis system. (A) Schematic comparison of the chemotaxis (Che) system of Escherichia coli to the WspR system of Pseudomonas and the WspH system of *Burkholderia*. The Che system modulates the direction of flagellar rotation in response to the binding of attractants to the receptor (e.g., serine and Tsr). The Wsp system is reported to respond to surface contact in P. aeruginosa, but the signal output varies in activating WspR (diguanylate cyclase) or WspH (function unknown). The panel on the right depicts sequence conservation of relative proteins among the Che, WspR, and WspH systems as presented numerically in Table S1 in the supplemental material. Dark green proteins show the greatest conservation, while light green proteins show the least conservation. CH_3_, methyl group; P, phosphate; CW/CCW, counterclockwise/counterclockwise. (B) The *wsp* genes in P. fluorescens Pf0-1 and B. cenocepacia HI2424 share synteny except that *wspR* is the terminal gene of a monocistronic operon and *wspH* is absent in the latter. *wspH* appears to be an independent transcription unit upstream from the *wsp* operon.

Studies of the Wsp signal transduction system in several Pseudomonas species have played an important role in establishing the positive correlation between cyclic di-GMP production and biofilm formation (17, 18, 27, 28, 32–37). The Pseudomonas Wsp system is encoded by the *wspABCDEFR* monocistronic operon that relays surface contact as an extracellular signal (38) to activate the diguanylate cyclase activity of WspR to produce cyclic di-GMP, which in turn stimulates EPS production and biofilm formation (5, 17–20). Currently, the functional model of the Wsp system (Fig. 1A) is largely based on sequence similarity to the respective Che proteins that collectively make up the enteric chemotaxis system (28). The transmembrane component of the Wsp complex, WspA, forms a trimer of dimers that is laterally distributed around the cell and acts to initiate cyclic di-GMP production (34). When activated by cellular contact with a surface, WspA is predicted to undergo a conformational change to expose its methylation sites, and a methyltransferase (WspC) and a methylesterase (WspF) ultimately determine the active state of WspA (18). Methylated WspA initiates the autophosphorylation of the histidine kinase WspE (27, 34, 37), which then phosphorylates the diguanylate cyclase WspR to activate cyclic di-GMP production. WspE also phosphorylates WspF, which terminates the signaling cascade (27, 34, 35). The Wsp complex is predicted to be functionally stabilized by WspB and WspD, which anchor the WspA trimer-of-dimer complex to WspE.

Although multiple studies have empirically demonstrated overarching functional parallelism in signal transduction between the Wsp and enteric chemotaxis systems (18, 27, 28, 34, 36, 39–42), fundamental gaps remain in our mechanistic understanding. For example, WspB and WspD are assumed to be functionally redundant as they are both homologous to CheW. However, this appears not to be the case since the subcellular localization of WspA differs between Δ*wspB* and Δ*wspD* mutants (18). Furthermore, all studies of the Wsp system had been limited to four Pseudomonas spp. until the discovery that Burkholderia cenocepacia HI2424 lacks the *wspR* gene and instead possesses *wspHRR* (here, *wspH*), a locus predicted to encode a unique hybrid histidine kinase/response regulator without the diguanylate cyclase domain (43, 44). Despite lacking WspR, mutations in the *wsp* genes of B. cenocepacia HI2424 produce wrinkly colony morphologies and SCV phenotypes similar to those of the *wsp* mutations in Pseudomonas spp. (43, 44). Although these observations imply that the Wsp system in B. cenocepacia HI2424 also functions to regulate cyclic di-GMP production, its cognate diguanylate cyclase, if any, remains unknown.

Since the initial characterization of the Wsp system in Pseudomonas fluorescens (27, 28, 32–34, 45), mutations within each of the *wsp* genes in other species have been reported to alter both the colony morphology and biofilm phenotypes, and they were either demonstrated or presumed to impact cyclic di-GMP production (18, 37, 39, 46, 47). Here, we show that mutations within the *wsp* operon of B. cenocepacia HI2424 do not alter the intracellular pool of cyclic di-GMP despite producing wrinkly colony phenotypes similar to those of comparable mutants in Pseudomonas spp. The function of the Wsp system may have further diverged beyond Pseudomonas and *Burkholderia* spp., but this remains entirely unexplored. To gain a broader appreciation of the mechanistic and functional robustness of Wsp systems, we first construct a data set of taxonomically diverse Wsp systems and assess their phylogenetic distribution and evolutionary history. We then evaluate sequence conservation at each amino acid residue to identify conserved genetic elements both within and outside annotated functional domains. By anchoring our sequence conservation analysis with diverse *wsp* mutations across multiple species that are known to impact signal transduction, we reevaluate the current functional model of the Wsp system and identify specific amino acid residues that likely manifest the key mechanistic differences.

## RESULTS AND DISCUSSION

### The WspR and WspH systems are functionally divergent.

The core signaling components of the enteric chemotaxis (Che) system and the Wsp system in Pseudomonas spp. and B. cenocepacia are expected to be mechanistically similar due to the high sequence conservation of the functional domains (Fig. 1A). However, the overall sequence homology among these three systems is relatively low, and the Wsp proteins between P. fluorescens Pf0-1 and B. cenocepacia HI2424 share greater homology (see Table S1 in the supplemental material). This suggests that the Wsp system in B. cenocepacia HI2424 functions similarly to the Pseudomonas Wsp system, but the former lacks WspR and is instead predicted to phosphorylate WspH, which lacks WspR’s GGDEF enzymatic domain required for cyclic di-GMP production (43, 44) (Fig. 1). To test if the Wsp system in B. cenocepacia HI2424 regulates cyclic di-GMP production and/or activates an unidentified cognate diguanylate cyclase, we collected comparable *wsp* mutants from P. fluorescens Pf0-1 and B. cenocepacia HI2424 that are known to impact signal transduction. The selected strains possess *wspA* mutations in the WspA/B/D/E signaling domain, *wspE* mutations in the receiver (REC) domain at the phosphoacceptor site, or *wspH*-*wspR* mutations in their respective REC domains. Mutations in *wspA* (V380A) and *wspE* (D648G) are known to increase cyclic di-GMP production and biofilm formation in Pseudomonas spp. (18, 32, 39). The B. cenocepacia HI2424 mutants utilized here, *wspA*(S258W) and *wspE* (D652N), were previously isolated and demonstrated to increase biofilm formation (43, 44). Conversely, a mutation in *wspR* (A113D) is predicted to terminate Wsp signaling as the mutated protein could no longer be phosphorylated (35). A mutation in *wspH* (L135F) has been shown to produce a smooth colony phenotype with defective biofilm formation (43), suggesting a reduced signaling output, if any at all.

**FIG 2:**
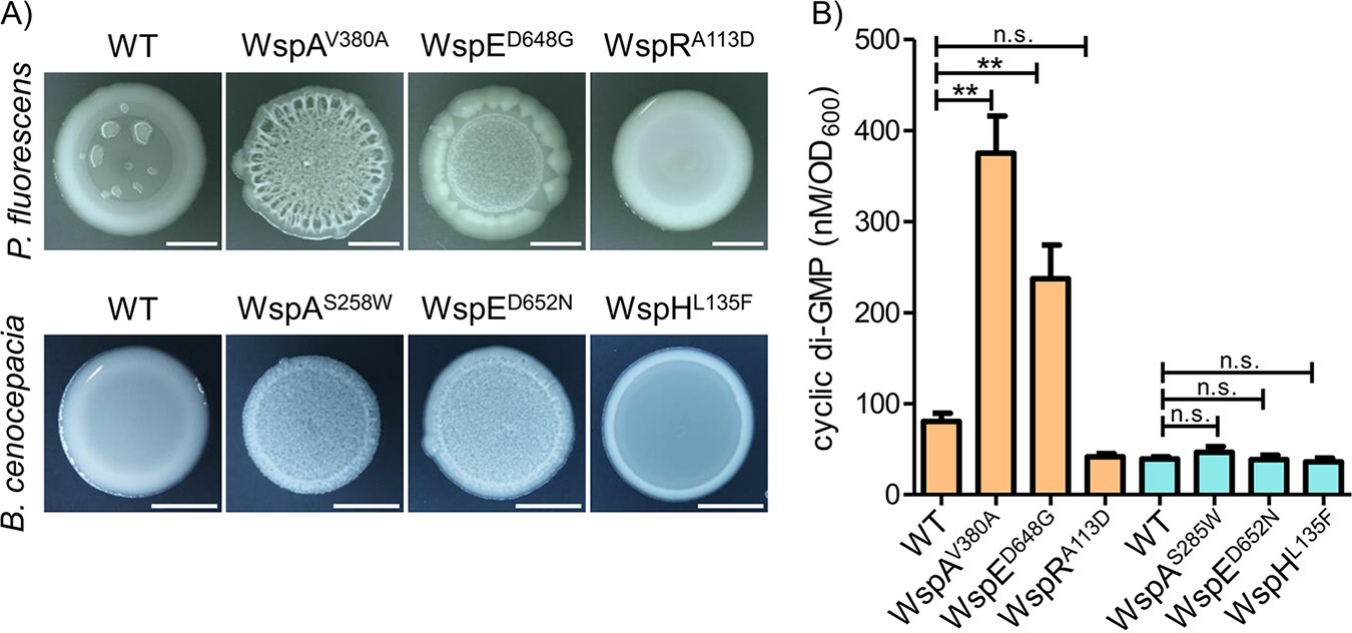
Comparable Wsp mutants of P. fluorescens and B. cenocepacia produce similar colony morphologies but differ in cyclic di-GMP production. (A) Colony images of P. fluorescens and B. cenocepacia (day 4) showing that mutations within the *wspA* or *wspE* genes in either organism produce the wrinkled phenotype, while mutations in *wspH* or *wspR* produce a smooth phenotype (bars = 5 mm). The mucoid patches observed in the WT P. fluorescens colony represent *rsmE* mutants that naturally emerge (79). (B) LC-MS/MS data showing that the wrinkly phenotype correlates with increased levels of cyclic-di-GMP in Wsp mutants in P. fluorescens (orange bars) but not in B. cenocepacia (teal bars). Plotted are the means from three replicates, and error bars represent the standard deviations. n.s. denotes no significant difference, and ** denotes a significant difference (*P* < 0.0001 by analysis of variance [ANOVA]; *P* < 0.01 by Tukey’s honestly significant difference [HSD] test).

We first assessed the colony morphologies of the *wsp* mutants for the iconic wrinkled phenotype that reflects increased cyclic di-GMP production across diverse species (18, 27, 28, 32–34, 37, 39, 45–47). Mutations in the *wspA* and *wspE* genes of both P. fluorescens Pf0-1 and B. cenocepacia HI2424 exhibit similar wrinkled phenotypes, while both the *wspR* mutant of P. fluorescens Pf0-1 and the *wspH* mutant of B. cenocepacia HI2424 exhibit smooth morphologies (Fig. 2A), as expected at low cyclic di-GMP levels. However, the *wspH* mutant appears to be less smooth or mucoid than the respective wild-type (WT) strains compared to the *wspR* mutant, which likely reflects functional differences between WspH and WspR. We next quantified the intracellular levels of cyclic di-GMP in the same set of mutants to test whether the altered colony morphologies indeed reflect increased cyclic di-GMP production. As predicted, cyclic di-GMP levels in *wspA* and *wspE* mutants of P. fluorescens Pf0-1 are significantly higher than those in the WT, unlike the *wspR* mutant (Fig. 2B). In contrast, none of the mutants of B. cenocepacia HI2424 significantly differ in cyclic di-GMP levels compared to the WT (Fig. 2B). This indicates that either the Wsp system in B. cenocepacia HI2424 does not regulate cyclic di-GMP production or its influence on cyclic di-GMP production is rapidly buffered. Regardless, there is a clear contrast here between the colony morphologies and cyclic di-GMP levels in the two species. Although the wrinkled colony morphology was positively correlated with biofilm production in these B. cenocepacia HI2424 *wsp* mutants (44), this appears to be achieved through a cyclic di-GMP-independent mechanism. This is not particularly surprising given that WspH lacks a diguanylate cyclase domain and instead possesses a hybrid histidine kinase domain. A recent *wsp* phenotype suppressor study in B. cenocepacia HI2424 implicated Bcen2424_1436 (RowR) as being critical for WspH signaling and subsequent polysaccharide synthesis (44). RowR is a predicted DNA-binding response regulator of an uncharacterized two-component transduction system; however, its direct interaction with WspH remains to be resolved.

Despite the overall sequence similarity, the Wsp system in B. cenocepacia HI2424 appears functionally divergent from the WspR system in Pseudomonas. However, comparable *wsp* mutations in B. cenocepacia HI2424 and clinically persistent Pseudomonas spp. converge on biofilm formation, producing similar clinically relevant phenotypes (22, 48, 49). To date, the WspH system has been reported only in B. cenocepacia HI2424 (43, 44), and its relative uniqueness remains unknown. Similarly, the shared characteristic synteny of the *wsp* operon has been described in only four Pseudomonas strains (27, 28, 33, 50), and the extent of its taxonomic distribution also remains unknown.

### Wsp systems are exclusive to the beta- and gammaproteobacteria, and the WspH system is restricted to *Burkholderia*.

To assess the phylogenetic distribution and evolutionary history of the Wsp system, we constructed a database of Wsp homologs from all publicly available bacterial genomes in GenBank (see Materials and Methods). Sequence similarity alone makes it difficult to bioinformatically distinguish Wsp homologs from those that function in chemotaxis (Fig. 1A) (51, 52). Fortunately, chemotaxis genes are infrequently located in a single operon (53, 54), in contrast to all annotated *wsp* genes (Fig. 1B). We thus identified syntenic Wsp homologs of P. fluorescens Pf0-1 and B. cenocepacia HI2424 with at least 30% sequence identity. Our analysis discovered 794 unique *wsp* gene clusters with conserved *wspA-wspF* synteny. Importantly, the use of the *wsp* genes from P. fluorescens Pf0-1 or B. cenocepacia HI2424 as independent queries generated overlapping results, indicating that synteny is a robust search parameter for identifying previously unannotated Wsp systems. All 794 identified *wsp* gene clusters were associated with either a *wspR* homolog (588) or a *wspH* homolog (206), as observed in P. fluorescens Pf0-1 or B. cenocepacia HI2424, respectively (Fig. 1B). We also found that no identified genome contains both H- and R-systems, and either system is present in a single instance per genome. For the sake of simplicity, we refer to *wsp* clusters with a *wspH* homolog as H-systems and those with a *wspR* homolog as R-systems.

**FIG 3:**
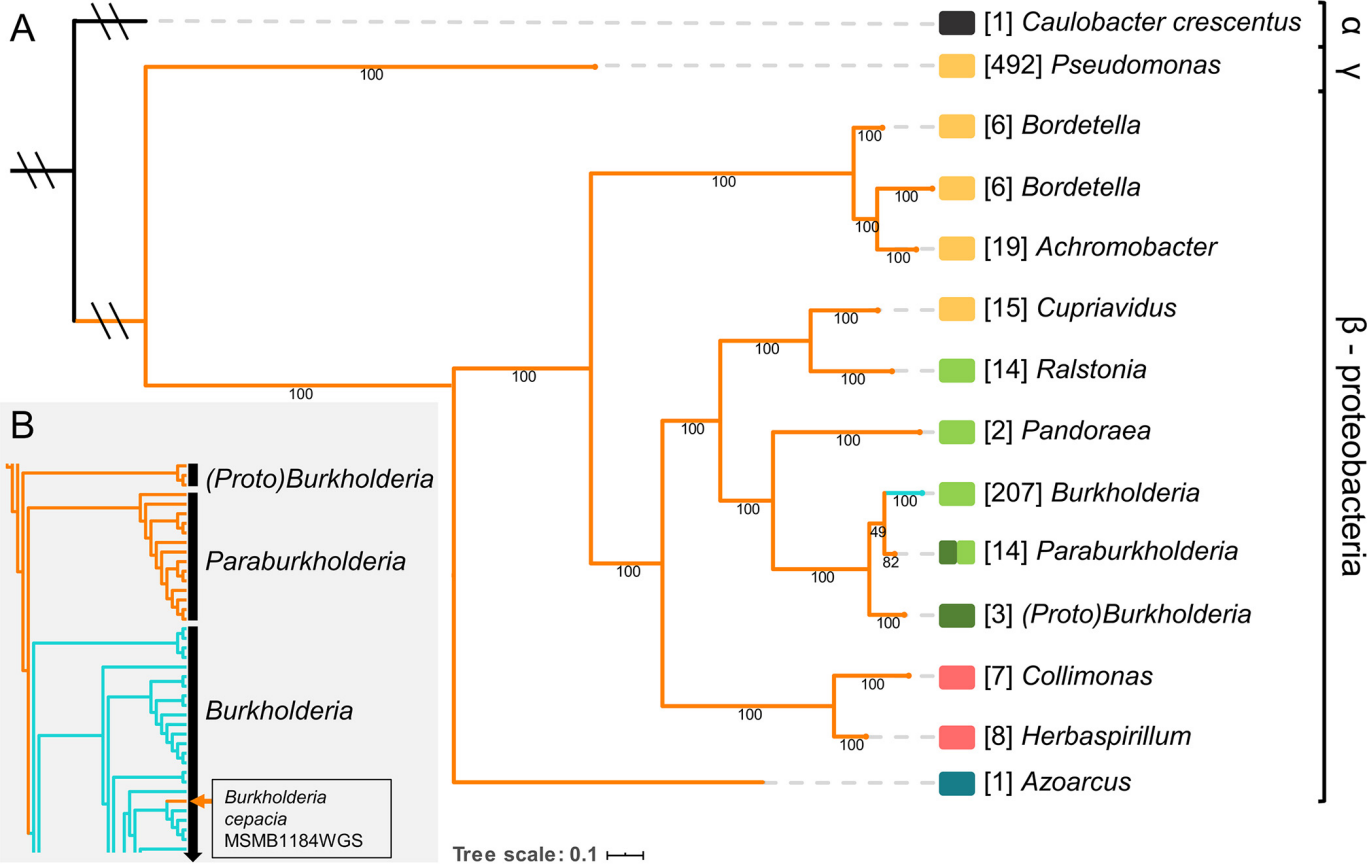
Phylogenetic analysis shows that the Wsp system is restricted to the beta- and gammaproteobacteria and that *wspH* is unique to *Burkholderia*. (A) Maximum likelihood species tree of organisms with the H-system (teal) or the R-system (orange). For simplicity, the tree was collapsed at the genus level, with the values in brackets indicating the number of strains in each branch. A total of 588 strains possess the R-system, and 206 strains possess the H-system, which is restricted to *Burkholderia*. (B) An expansion of the *Burkholderia-Paraburkholderia* subgroup shows that only one strain (Burkholderia cepacia MSMB1184WGS) possesses the R-system. An independent phylogenetic assessment of Wsp proteins identified five unique Wsp system clades (see Fig. S1 in the supplemental material): blue, clade 1; yellow, clade 2; pink, clade 3; light green, clade 4; dark green, clade 5. Values reported under individual branches are bootstrap support values (out of 100).

We next constructed a phylogenetic tree to visualize the taxonomic distribution of the H- and R-systems, which reveals that they are present exclusively in beta- and gammaproteobacteria (Fig. 3A). The R-system is distributed across both the beta- and gammaproteobacteria, while the H-system is limited to *Burkholderia*. The 12 *Bordetella* strains in our data set form two distinct clades, and three *Burkholderia* strains branch out earlier from the remaining *Burkholderia* and *Paraburkholderia* clades. These three strains are currently labeled as *Burkholderia* in the NCBI database, but here, we refer to them as “(*Proto*)*Burkholderia*” as their true taxonomic identities remain unresolved. (*Proto*)*Burkholderia* possesses the R-system, as does *Paraburkholderia*, suggesting that the R-system predates the H-system found exclusively in *Burkholderia*. Further expanding the *Burkholderia* clade shows that the R-system is present in only one strain (Fig. 3B), Burkholderia cepacia MSMB1184WGS, a soil isolate from the Australian Northern Territory. Pseudomonas is the lone genus representing the gammaproteobacteria, indicating that it likely acquired the R-system through horizontal gene transfer.

### Phylogenetic incongruence reflects multiple horizontal transfer events of the R-system.

**FIG 4:**
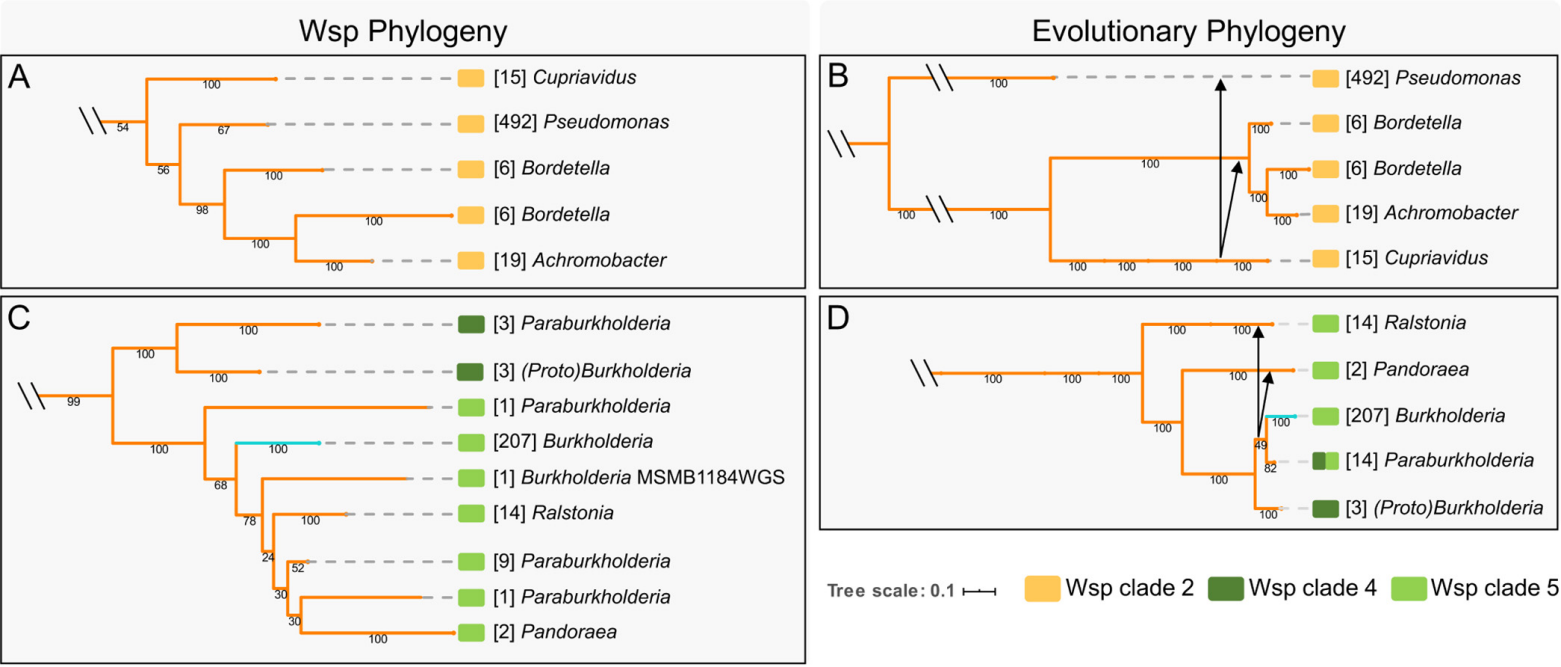
Phylogenetic analysis of the core Wsp proteins indicates multiple horizontal gene transfer events and establishes the R-system as the predecessor to the H-system. Shown here is a trimmed version of the Wsp phylogeny (A and C) for Wsp clades 2, 4, and 5 adjacent to the evolutionary phylogeny (B and D). The H-system (teal) and R-system (orange) Wsp phylogenies were constructed using the amino sequences of the core Wsp proteins (WspA, WspB, WspC, WspD, WspE, and WspF) and are detailed in Fig. S1 in the supplemental material. Clades identified in Fig. S1 are reported as color blocks as indicated at the bottom. Values reported under branches are bootstrap support values (out of 100), and those in brackets represent the total number of genomes/Wsp systems. Phylogenetic incongruences are highlighted by black arrows, representing the direction of each horizontal transfer event. The R-system from *Cupriavidus* or its ancestor was likely transferred to Pseudomonas, *Bordetella*, and *Achromobacter* (B), and the R-system from the common ancestor of *Burkholderia* and *Paraburkholderia* likely transferred to *Ralstonia* and *Pandoraea* (D).

We evaluated potential horizontal transfer events of the Wsp system by assessing the incongruence between the species and Wsp phylogenies (55, 56). A phylogeny of the 794 Wsp systems was constructed using the concatenated peptide sequences of the core Wsp signaling proteins (WspA, WspB, WspC, WspD, WspE, and WspF), which diverges into five distinct clades (Table S2 and Fig. S1). The sequence variations in the Wsp signaling core alone clearly differentiate the H- and R-systems even in the absence of WspH and WspR. The significant differences between the species (Fig. 3) and Wsp (Fig. S1) trees strongly suggest that the Wsp system has been subjected to multiple horizontal transfer events, as summarized in Fig. 4.

We observe that the R-system likely originated in *Azoarcus* (clade 1) and then radiated throughout the betaproteobacteria and into Pseudomonas (Fig. S1), the sole member of the gammaproteobacteria (Fig. 3). In clade 2, the R-systems of Pseudomonas, *Bordetella*, and *Achromobacter* share a node with *Cupriavidus* (Fig. 4A), yet these genera are taxonomically distinct (Fig. 4B). This observation, as supported by the high bootstrap values in both phylogenies, indicates that *Cupriavidus* or its ancestor served as the common source for the horizontal transfer of the R-system into the phylogenetically distant Pseudomonas, *Bordetella*, and *Achromobacter* genera (Fig. 4B). Interestingly, each genus in clade 2 comprises opportunistic pathogens that are frequently associated with the respiratory environment. The R-systems in clades 4 and 5 of the remaining betaproteobacteria appear to share an ancestry (Fig. 4C) but show that the R-system from the ancestor of *Burkholderia* and *Paraburkholderia* likely transferred horizontally into *Ralstonia* and *Pandoraea* (Fig. 4D). This is clearly observed in the phylogenies where *Ralstonia* and *Pandoraea* taxonomically diverged before *Burkholderia* (Fig. 4D), but the acquisition of their Wsp systems occurred after *Burkholderia* (Fig. 4C). Interestingly, the Wsp system of *Paraburkholderia* is not confined to a single node in the Wsp phylogeny and is instead scattered among *Ralstonia*, *Pandoraea*, and *Burkholderia* (Fig. 4C). This indicates that the Wsp systems of *Paraburkholderia* have high sequence variation, which could manifest unique functions such as responding to diverse stimuli or interacting with other response regulators.

Much like the taxonomic phylogeny, the H-system in *Burkholderia* forms a monophyletic clade, indicating that the signaling core of the H-system is highly conserved but is distinct from the R-system. The unique presence of the R-system in B. cepacia MSMB1184WGS presents an interesting case. Our analysis suggests that the H-system of this strain was independently replaced with an R-system after the radiation of the H-system in *Burkholderia* (Fig. 4C). Another possibility is that the R-system of this strain is related to the original source for the evolution of the H-system. Although many of the organisms in our data set are associated with respiratory infections, the Wsp systems are present in both opportunistic and nonpathogenic species. This suggests that horizontal transfer events of Wsp predate adaptations to the human host and that individual Wsp systems have functionally diverged beyond the emergence and integration of the *wspH* gene.

### Sequence conservation of Wsp systems and acquired mutations converge on predicted functional domains and unannotated regions.

**FIG 5:**
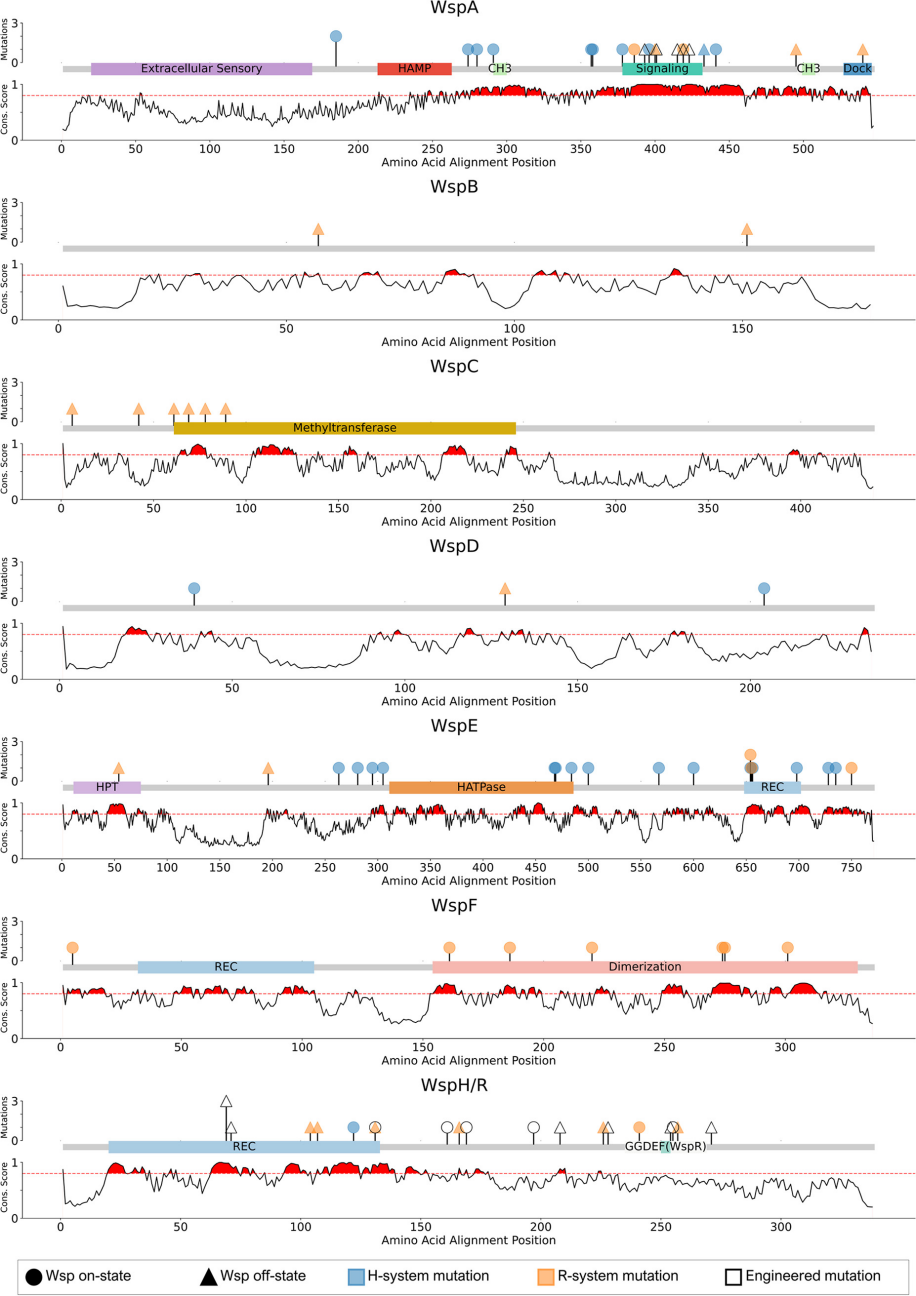
Evaluating the conservation of Wsp proteins identifies key residues that fall within and outside annotated functional domains. The 794 peptide sequences for each protein were used to generate individual alignments. Annotation data are derived from NCBI CDD (Conserved Domain Database), Prosite, or Che homology as indicated in Table S4 in the supplemental material. Reported naturally occurring missense mutations from the literature, engineered missense mutations reported in the literature, mutations that turn on the respective Wsp systems, and those that turn off the system are indicated. The *y* axis represents the Shannon entropy evaluation for each protein alignment, where weighted values near 1 indicate high sequence conservation and values near 0 indicate weak sequence conservation. Regions where the weighted Shannon entropy metric equals or exceeds 0.8 are shaded in red and denote regions likely to have functional or structural importance. Many of the previously identified mutations reside in these regions of high conservation, but the functional role of these residues remains unknown.

Missense mutations in *wsp* genes that impact biofilm formation are commonly identified in clinical isolates and experimental evolution studies (18, 27, 28, 32–34, 37, 39, 45–47). Such mutations likely occur in key functional residues, but the Wsp system as a whole remains poorly annotated. We thus aligned the amino acid sequences of all Wsp proteins in our data set to generate a consensus sequence and annotated functional domains based on homology to the enteric chemotaxis (Che) system and empirical studies of the Pseudomonas Wsp system (Tables S3 and S4). We then assessed the 794 sequence alignments for conservation using a weighted Shannon entropy algorithm where a conservation score near 1 indicates high conservation and a conservation score near 0 indicates weak conservation (57). Regions exceeding a conservation score of 0.8 are highlighted in red since this threshold reliably captures known functional residues (57). Finally, we compiled all *wsp* missense mutations from the literature that have been predicted to impact the signaling of the respective Wsp systems and then mapped them to our consensus sequence (Table S3). We summarize the sequence conservation, newly annotated functional domains, and sites of missense mutations in Fig. 5 and the sequence conservation profiles of H- and R-systems independently in Fig. S2. The H-systems share consistently higher sequence conservation across all Wsp proteins, which reflects their phylogenetic isolation (Fig. S2). In general, our results complement previous reports and speculations on Wsp function but also reveal surprising patterns.

WspA exhibits strikingly high sequence conservation across four distinct regions. Reflecting on the significance of the trimer-of-dimer interaction (signaling) domain of WspA (18), the corresponding region is extremely conserved. This particular region also shares 74.5% sequence similarity to the enteric chemotaxis Tsr signaling domain (58, 59), and we found that the signaling domain in WspA likely extends beyond those reported (18) to include residues 378 to 432 (Table S4). Mutations within this signaling domain likely alter the stability of the trimer-of-dimer interactions, resulting in either increased or decreased signaling to WspE (18). Two additional regions exhibit high conservation (residues 291 to 300 and 499 to 508), which we predict to function as the methylation sites (CH_3_) by WspC (Table S4). Although the methylation of WspA has never been experimentally confirmed (51), high sequence conservation coupled with homology to the chemotaxis system strongly suggests that these sites are likely methylated. Finally, the chemotaxis Tsr signaling domain contains a docking site for the methyltransferase CheR (Fig. 1A), which is present as the last 5 residues of the Tsr C terminus (60). Although WspA does not contain the same motif, we observe high conservation of the last 9 amino acids (aa) (aa 536 to 545) at the C terminus. This region likely represents the docking site for WspC and WspF. Interestingly, there is relatively low sequence conservation in the extracellular sensory domain (Fig. 5). The same pattern holds across the R-system, but the H-system shows much higher levels of conservation (Fig. S3). These results suggest that these two systems may respond to unique extracellular stimuli, which could also apply across the R-system as well given the extent of sequence variations observed.

WspB and WspD are loosely thought to function to relay the signal between WspA and WspE, like their chemotaxis CheW homolog (61, 62) (Fig. 1A). However, O’Connor et al. found that deletions of *wspB* and *wspD* in P. aeruginosa manifest discrete outcomes on the subcellular localization of WspA (18). A Δ*wspD* strain caused WspA to become polarly localized, dramatically reducing WspR phosphorylation. In contrast, a Δ*wspB* strain had little effect on WspA’s naturally lateral presence but resulted in similarly reduced WspR phosphorylation. This indicates that WspB and WspD are not functionally redundant and suggests an essential role for WspD in modulating the subcellular localization of the Wsp system and the intracellular pool of cyclic di-GMP. Our analysis reveals that WspB and WspD are the least conserved among all the Wsp proteins, and they exhibit unique conservation signatures (Fig. 5 and Fig. S3). We identified six regions of WspB where motifs between 8 and 17 residues in length are highly conserved. WspD similarly has 7 motifs ranging between 5 and 19 residues. Comparisons of these motifs reveal that they are unique to their respective proteins and have no known or predicted function. Although we were unable to bioinformatically predict functional domains in either protein, their unique conservation signatures described here likely represent sites for interacting with WspA and WspE (Fig. 1A) and potentially with other non-Wsp proteins to modulate subcellular localization. Interestingly, no mutation has ever been reported in the *wspB* gene for the H-system (Fig. 5).

Both the conserved regions and reported mutations in WspC and WspF largely associate with our annotated domains (Fig. 5). WspC is predicted to function as the main activator of the Wsp system, and WspF acts as the repressor (Fig. 1A). Consequently, *wspF* mutations from the literature exclusively act to turn on the Wsp system, while mutations in *wspC* exclusively turn off the Wsp system (Fig. 5). However, there is a striking pattern here where no mutation has ever been reported for either gene of the H-system, in sharp contrast to 25% of all R-system mutations falling within *wspC* or *wspF* (Table S3). This is surprising given that WspC and WspF are predicted to function as the main switch of the Wsp system. Sequence conservation patterns suggest that WspC and WspF indeed function to methylate and demethylate WspA, as predicted. Although we do not expect our compiled mutational or experimental evolution data to be saturating for any *wsp* gene, the absence of mutations in *wspC* or *wspF* genes of the H-system to date leads to the prediction that the methylation state of WspA may have a reduced influence on the activity of the H-system compared to the R-system.

We observe high conservation in the REC domain (aa 648 to 702) of WspE (Fig. 5) and mutations within specific residues that appear to activate WspE in a WspA-independent manner (17). The HATPase domain shows high conservation, which is expected as this region is essential for binding to ATP and ultimately phosphorylating WspF and WspR/WspH. We observe high conservation in unannotated regions that flank the HATPase domain that are frequently mutated in the H-system but entirely unaffected in the R-system. Given that WspE interacts with either WspR or WspH, this particular region may be uniquely important for the H-system. It is likely that these WspE-activating mutations observed exclusively in the H-system manifest a conformational change that initiates HATPase function in the absence of a stimulus. The region between the histidine phosphotransfer (HPT) and HATPase domains in the chemotaxis protein CheA constitutes the P2 and P3 domains, which are responsible for CheY (response regulator) and CheB (methylesterase) docking and CheA dimerization, respectively (Fig. 1A). However, BLAST assessments reveal no significant similarities between WspE and CheA for these regions, and therefore, WspE lacks this annotation. It is possible that the H-system mutations in the 3′-adjacent HATPase region may affect either WspE dimerization or WspH/WspF docking, and this may be uniquely important for the H-system.

Previous work compared WspH of B. cenocepacia and WspR of P. aeruginosa to find ∼70% sequence similarity within the REC domain (43), suggesting that WspH and WspR are similarly phosphorylated by WspE. However, WspH and WspR differ substantially in their C termini, where WspR contains the diguanylate cyclase GGDEF domain and WspH possesses a histidine kinase speculated to phosphorylate an unknown response regulator (43). We observe comparably high conservation in our large data set within the WspH/R REC domain (Fig. 5). As expected, we observe weak conservation of this C terminus in our WspH/R alignment and greater conservation when H- and R-systems are compared independently (Fig. S2). Interestingly, only the GGDEF region of the WspR C terminus exhibits high conservation.

### Identification of residues that are uniquely conserved between H- and R-systems.

Among the 2,899 consensus amino acid residues of the H- and R-systems, we have identified 43 residues that are likely important for the specialized function of the H-system (see Materials and Methods). These 43 residues are highly conserved in both H- and R-systems but also unique to each system (Table 1) and represent nonconservative substitutions that occurred within the H-system (63). Selective pressures have forced nearly all H-systems to retain these residues, suggesting that they are essential to H-system signaling. Notably, 23 of the 43 identified residues are in the C terminus of WspH, which does not contain an enzymatic domain like WspR but is instead a histidine kinase speculated to phosphorylate an unknown response regulator that stimulates biofilm production (43). Seven residues occur in the REC domain of WspH, which is predicted to interact with and become phosphorylated by WspE. WspE of the H-system has three substitutions, with two occurring in the HPT relay domain and one in an unannotated region of the C terminus. We predict that these residues are unique to WspE and WspH interactions and WspH phosphorylation. Five substitutions were identified in unannotated regions of WspA, WspB, and WspD; four remaining substitutions were within the methyltransferase domain of WspC; and none were detected in WspF. These uniquely conserved substitutions likely differentiate the signaling mechanism of the H-system from that of the R-system, which remains entirely unexplored.

**TABLE 1:** Assessment of unique conservation between the H- and R-systems identifies residues important for the specialized function of the H-system

Locus	Residue position^*a*^	Domain^*b*^	R-system residue^*c*^	R-system conservation score	H-system residue^*c*^	H-system conservation score
WspA	360		L	0.80182	S	0.99999
WspA	484		V	0.82324	F	0.9892
WspA	516		V	0.88076	A	0.99999
WspB	134		Y	0.82288	W	0.93129
WspC	68	Methyltransferase	A	0.83557	L	0.98174
WspC	69	Methyltransferase	V	0.86569	F	0.99208
WspC	108	Methyltransferase	L	0.85525	A	0.98103
WspC	119	Methyltransferase	I	0.84741	A	0.94557
WspD	139		C	0.81521	A	0.94529
WspE	70	HPT	H	0.86267	G	0.98887
WspE	71	HPT	V	0.81219	R	0.981
WspE	300		A	0.82943	Q	0.99921
WspH/R	19	REC	M	0.86114	S	0.95937
WspH/R	28	REC	M	0.87022	F	0.98196
WspH/R	32	REC	A	0.92994	V	0.99999
WspH/R	84	REC	Y	0.83043	W	0.97545
WspH/R	92	REC	D	0.82661	R	0.91437
WspH/R	108	REC	S	0.85009	R	0.93589
WspH/R	112	REC	A	0.86297	V	0.83238
WspH/R	137		R	0.93451	N	0.96292
WspH/R	149		Y	0.95783	L	0.98218
WspH/R	150		R	0.91456	D	0.98218
WspH/R	151		A	0.95059	F	0.98218
WspH/R	153		R	0.95311	S	0.99999
WspH/R	155		S	0.9542	D	0.97478
WspH/R	156		Q	0.9542	M	0.97478
WspH/R	168		R	0.89453	A	0.99136
WspH/R	170		M	0.82239	A	0.99999
WspH/R	171		N	0.84907	L	0.99999
WspH/R	176		T	0.96372	I	0.99136
WspH/R	177		G	0.94363	H	0.94821
WspH/R	213		K	0.9026	W	0.97741
WspH/R	215		Y	0.86625	K	0.90022
WspH/R	216		N	0.88655	A	0.89947
WspH/R	221		H	0.89829	S	0.83583
WspH/R	226		E	0.81682	R	0.97193
WspH/R	227		A	0.81562	M	0.99166
WspH/R	247		A	0.94744	C	0.85349
WspH/R	249		Y	0.84751	L	0.83019
WspH/R	270		A	0.82674	P	0.97357
WspH/R	298		G	0.82934	V	0.99818
WspH/R	325		K	0.84267	L	0.91421
WspH/R	328		G	0.85937	P	0.88478

a Reflects the amino acid position within the H-system and R-system alignments where the identified residue is highly conserved in its respective alignment but represents a nonconservative mutation when compared.

b Domain evidence can be found in Table S4 in the supplemental material. HPT, histidine phosphotransfer; REC, receiver.

c The amino acid at the specified position within the R-system and H-system alignments is highly conserved as reflected by the R-system and H-system conservation scores.

### Conclusion.

This study presents a broad phylogenetic analysis of the Wsp signaling system to expand our current understanding of the Wsp signaling system, which had thus far been restricted to four Pseudomonas spp. and, more recently, B. cenocepacia HI2424. Ironically, we have found that Pseudomonas spp. are the only gammaproteobacteria to possess the Wsp system, which appears to have originated in the betaproteobacteria, with strong indications of multiple horizontal gene transfer events. There is clear genetic and biochemical evidence (18, 27, 28, 32–34, 37, 39, 45–47) to suggest that the core signaling components of the Wsp system in Pseudomonas mechanistically function much like those of the enteric chemotaxis system. Our sequence conservation and mutation analyses extend our current understanding of Wsp to include more evolutionarily divergent organisms and reveal the strong potential for mechanistic diversity among individual Wsp systems.

All Wsp systems appear to utilize either WspH or WspR exclusively as the terminal output of the signaling cascade, and all organisms possess only one H- or R-system. A previous study discovered the H-system in B. cenocepacia HI2424 to show that WspH lacks the diguanylate cyclase GGDEF domain found in WspR and speculated that WspH likely activates a unique diguanylate cyclase (43). This was based on the observation that various mutations in *wsp* genes caused the hallmark wrinkly colony morphology and increased biofilm formation associated with elevated cyclic di-GMP production in diverse bacterial species. However, our study clearly demonstrates that these *wsp* mutations in B. cenocepacia HI2424 do not alter cyclic di-GMP production, in contrast to comparable *wsp* mutations in P. fluorescens Pf0-1. Despite the striking difference in the functional outputs of the H- and R-systems, they share high levels of sequence conservation within and beyond key functional domains that overlap the enteric chemotaxis system. One major difference that we observed was the complete absence of reported mutations in *wspC* and *wspF* genes of the H-system, in contrast to those of the R-system. We also identified 43 highly conserved amino acid residues across all Wsp proteins that are uniquely modified in the H-system. These specific residues likely differentiate mechanistic variations between the H- and R-systems, and we suspect that the methylation state of WspA exerts a relatively reduced role in the signaling cascade of the H-system compared to the R-system.

The Wsp proteins of the R-system exhibit greater overall sequence variation than those of the H-system. This is not surprising since the H-system is phylogenetically restricted to *Burkholderia* spp. and the R-system is much more divergent. However, nearly all of the annotated functional domains are highly conserved across the R-system, with the exception of the extracellular sensory domain of WspA. The external stimulus of the Wsp system long remained a mystery until a recent study demonstrated surface contact to be the main stimulus in P. aeruginosa (38). The relatively large sequence variation observed within this extracellular sensory domain indicates strong potential for independent adaptations to diverse external stimuli. We found that WspB and WspD proteins exhibit the smallest amount of sequence conservation, yet we did not observe any instance of a *wsp* operon lacking either of these proteins, strongly indicating that they are both functionally important. In contrast to the enteric chemotaxis system, which utilizes CheW to physically bridge the methylation and phosphorylation signaling modules, the Wsp system is thought to utilize both WspB and WspD in an analogous manner. However, there is clear evidence in P. aeruginosa that WspB and WspD are not functionally redundant (18), and we observed distinct sequence conservation patterns between these two proteins. We also found that all mutations reported in WspB and WspD proteins act to deactivate the R-system, while mutations in WspD of the H-system exclusively act to turn it on. Furthermore, no mutations have been reported for WspB of the H-system. Given the tremendous influence of WspD on P. aeruginosa’s Wsp signaling cascade (18), these so-called accessory proteins likely play both functionally important and diverse roles among individual Wsp systems.

Mutations in *wsp* genes are frequently identified in clinical specimens and are associated with increased biofilm formation (22, 24, 25, 64–69), whether or not this is achieved through elevated production of cyclic di-GMP. Genetically deregulating the Wsp system appears to be a significant but transient adaptive strategy in a dynamic and overcrowded ecosystem. Our study shows that leaning on functional analogies to the enteric chemotaxis system serves as both a valid and critical foundation for future studies to understand the functional blueprint of diverse Wsp systems in greater detail. Key focus should be placed on elucidating the function of WspH and its interacting partners, disentangling the functional uniqueness of WspB and WspD, and identifying extracellular stimuli for WspA in diverse organisms.

## MATERIALS AND METHODS

### Strains and culture conditions.

All bacterial strains were routinely grown in Lennox LB (Fisher BioReagents) or on Pseudomonas agar F (PAF) (Difco). Pseudomonas F (PF) broth (a nonagar variant of PAF) was prepared with the following composition: pancreatic digest of casein at 10 g/liter (Remel), proteose peptone no. 3 at 10 g/liter (Remel), dipotassium phosphate at 1.5 g/liter (Sigma-Aldrich), and magnesium sulfate at 1.5 g/liter (Sigma-Aldrich). Pseudomonas and *Burkholderia* strains were incubated at 30°C and 37°C, respectively, with shaking at 250 rpm when applicable. *wsp* mutants used in this study were previously isolated from Burkholderia cenocepacia HI2424 (43) and Pseudomonas fluorescens Pf0-1 (39). Colony morphology was evaluated in triplicate by inoculating PAF plates (9-mm petri dish with 25 ml of PAF) with 25 μl of a culture grown overnight and then incubating the plates at 22°C for 4 days.

### Cyclic di-GMP extraction and LC-MS/MS.

An isolated colony of each strain was transferred to PF broth and grown overnight at the respective temperatures. Cultures grown overnight were diluted to an optical density at 600 nm (OD_600_) of 0.04 in triplicate, incubated at the respective temperatures for 2 to 4 h until the samples reached an OD_600_ of 0.5, and then promptly processed for cyclic di-GMP extraction. Cyclic di-GMP extraction and quantification procedures were performed according to the protocols established by the Michigan State University Research Technology Support Facility (MSU-RTSF): the MSU_MSMC_009 protocol for dinucleotide extractions and the MSU_MSMC_009a protocol for liquid chromatography-tandem mass spectrometry (LC-MS/MS). All of the following steps of cyclic di-GMP extraction were carried out on ice unless noted otherwise. One milliliter of culture at an OD_600_ of 0.5 was centrifuged at 15,000 relative centrifugal force (RCF) for 30 s, and cell pellets were resuspended in 100 μl of ice-cold acetonitrile-methanol-water (40:40:20 [vol/vol/vol]) extraction buffer supplemented with final concentrations of 0.01% formic acid and 25 nM cyclic di-GMP-fluorinated internal standard (cyclic di-GMP-F) (catalog no. 1334145-18-4; InvivoGen). Pelleted cells were mechanically disturbed with the Qiagen TissueLyser LT system at 50 oscillations/s for 2 min. Resuspended slurries were incubated at −20°C for 20 min and pelleted at 15,000 RCF for 15 min at 4°C. The supernatant was transferred to a prechilled tube and then supplemented with 4 μl of 15% (wt/vol) ammonium bicarbonate (Sigma-Aldrich) buffer for stable cryo-storage (47). Extracts were stored at −80°C for less than 2 weeks prior to LC-MS/MS analysis at MSU-RTSF. We observed sample degradation with repeated freeze-thaw cycles, so we ensured that the extracts were never thawed prior to LC-MS/MS analysis. All samples were evaporated under a vacuum (SpeedVac, no heat) and redissolved in 100 μl of the mobile phase (10 mM tributylamine [TBA]–15 mM acetic acid in water-methanol [97:3, vol/vol] [pH 5]). Ultrahigh-performance LC-MS/MS (UPLC-MS/MS) quantification was performed using a Waters Xevo TQ-S instrument with the following settings: cyclic di-GMP-F at an *m/z* transition of 693 to 346 with a cone voltage of 108 and a collision voltage of 33 and cyclic di-GMP at an *m/z* transition of 689 to 344 with a cone voltage of 83 and a collision voltage of 31. Cyclic di-GMP data were normalized to 25 nM cyclic di-GMP-F by MSU-RTSF to account for sample matrix effects and sample loss during preparation. We used the OD_600_ measurements of the initial samples to report the quantified cyclic di-GMP as nanomolar/OD unit and visualized the results with GraphPad Prism5.

### Generation of the H- and R-system data set.

The WspH-encoding system of B. cenocepacia HI2424 and the WspR-encoding system of P. fluorescens Pf0-1 were used as queries to search the BCT bacterial subdivision of GenBank for syntenic homologs (NCBI protein accession no. ABK10522.1, ABK10523.1, ABK10524.1, ABK10525.1, ABK10526.1, ABK10527.1, ABK10528.1, and ABK10529.1 and accession no. ABA72795.1, ABA72796.1, ABA72797.1, ABA72798.1, ABA72799.1, ABA72800.1, and ABA72801.1, respectively). All previous Wsp studies show that the *wsp* operon is highly syntenic (18, 27, 28, 32–34, 37, 39, 45–47), and B. cenocepacia HI2424 shares this synteny (43). Cooper et al. identified that *wspH* had relocated from the 3′ end to the 5′ end of the WspH gene cluster (43). To determine if syntenic homologs have a similar *wspH* gene cluster arrangement and to generate a large data set of H- and R-systems, we used MultiGeneBlast v-1.1.14 to search GenBank and identify homologous Wsp systems. MultiGeneBlast performs a BLAST analysis for query sequences and then provides a weighted score based on the synteny of the genes (70). We executed MultiGeneBlast locally against the bacterial subdivision of GenBank (BCT subdivision) and reported the first 2,000 syntenic homolog hits for the WspH-encoding system of B. cenocepacia HI2424 and the WspR-encoding system of P. fluorescens Pf0-1. The P. fluorescens Pf0-1 search results are provided in Data Set S1 at https://dsc.duq.edu/biology-data/4/, and the B. cenocepacia HI2424 search results are provided in Data Set S2. Our search produced 1,640 R-system hits and 1,500 H-system hits. Both searches generated fewer than 2,000 hits as we had specified, indicating that we had identified nearly all available *wsp* homologs in the GenBank database that match our queries.

Assessment of the R-system data set showed that the first 810 R-system hits possessed a GGDEF or GGEEF domain and shared synteny with the P. fluorescens Pf0-1 queries. Hits 811 and 812 of the R-system search identified species that did not contain a *wspR* response regulator hit because an entry for that locus was not included in their GenBank annotation files. Hits 813 to 827 and 829 identified *Burkholderia* spp. with a 5′ response regulator that lacked a GGDEF or GGEEF domain and closely resembled the *wspH* gene cluster of B. cenocepacia HI2424. Hits 830 and above did not contain a gene cluster with similarity to the *wspR* or *wspH* gene sets. Based on this information, we collected the first 810 R-system hits to be included in the final R-system data set. A similar distribution was observed for the 1,500 H-system hits. The first 217 H-system hits identified were *Burkholderia* species with a 5′ response regulator that lacked a GGDEF or GGEEF domain. Hits 218 to 818 included a GGDEF or GGEEF domain and closely resembled the *wspR* gene cluster of P. fluorescens Pf0-1. Hits 819 to 829 were Pseudomonas species that did not contain a *wspH* hit. Manual analysis identified these as *wspR*-containing operons with low *wspH* homology. Hits 830 and above did not contain a gene cluster with similarity to the *wspR* or *wspH* gene sets. Based on this information, we collected the first 818 H-system hits to be included in our H-system data set. Compiling the two data sets and removing duplicate hits generated a final data set of 794 total hits. We identified 588 R-systems based on the presence of the GGDEF or GGEEF domain and 206 H-systems that lacked this domain and were 5′ adjacent. We did not identify any H-systems with a 3′ response regulator, nor did we find any organisms that contained both H- and R-systems.

### Phylogenetic analysis of WspH/R data set strains.

The MultiGeneBlast output includes the NCBI organism accession number and the protein accession numbers for each sequence identified in the analysis. The RefSeq URLs for the assembly statistics file for each organism were determined from the NCBI organism accession numbers using the Entrez.esearch and Entrez.read utilities of BioPython v-1.78 (71) (see File S1 at https://dsc.duq.edu/biology-data/4/). Data for Caulobacter crescentus (see Table S2 in the supplemental material) was obtained manually for rooting the species phylogeny. We chose C. crescentus because it is an alphaproteobacterium that contains the diguanylate cyclase PleD, which has been previously used as a reference in *wspR* studies (41). R statistical software was then used to download all RefSeq assembly data files for each organism using generated URLs based on the assembly statistics files (code provided in Files S2 to S4 at https://dsc.duq.edu/biology-data/4/). The obtained files were compared to the Bac120 ubiquitous housekeeping gene set established previously by Parks et al. (72). This search was conducted with the hmmsearch tool of HMMER v-3.3.2 with an E value cutoff of 1e−10 (73). The hidden Markov model (HMM) files were converted to sequence files using the esl.sfetch tool of the Easel HMMER library using default settings. Some organisms contained multiple copies of a housekeeping gene. In these instances, the gene with the lowest E value was selected for the analysis. Organisms that lacked any of the Bac120 genes received a blank fasta entry, indicated by dashes. Peptide-encoding sequences for each gene in the Bac120 set were aligned using MAFFT v-7.475 with 1,000 iterations and global pair options (74). Trimal v-1.3 was then used to remove gaps with a 0.9 cutoff value option (75). This generated 120 individual alignments with 795 organisms per alignment (794 from the data set plus the rooting sequence). Finally, one continuous sequence was generated for each organism by concatenating the 120 aligned sequences into a single string. This ultimately generated a single file with 795 organisms where one entry included the aligned information for all 120 genes. This method has a much higher sensitivity to differentiate species than traditional 16S phylogenetic methods (72). The final alignment was evaluated under the maximum likelihood (ML) algorithm RAxMLv-8.0.0 (HPC-HYBRID-AVX2) with the PROTCATBLOSUM62 model, 100 bootstraps, and the -f a hill-climbing options enabled. Final trees were rendered using the ITOL software suite (Fig. 3).

### Phylogenetic analysis of the Wsp signaling core.

MultiGeneBlast provides protein accession numbers for sequences identified in the analysis. Sequence files for these peptides were downloaded from the NCBI with the provided accession numbers using the Entrez.esearch, Entrez.read, and Entrez.fetch utilities of BioPython (71) (code provided in File S1 at https://dsc.duq.edu/biology-data/4/). As in the species tree, C. crescentus was selected to root the tree. P. fluorescens Pf0-1 *wsp* homologs were identified in C. crescentus and are described in Table S2. fasta files were independently generated for WspA, WspB, WspC, WspD, WspE, or WspF, resulting in 795 sequences per file (794 from the data set plus the rooting sequence). WspR and WspH were omitted from this analysis since they are divergent in both function and sequence. The fasta files were aligned using MAFFT with 1,000 iterations and global pair options (74). Trimal was then used to remove gaps with a 0.3 cutoff value option (75). A continuous “Wsp signaling core” sequence was generated for each organism by concatenating the six independently aligned Wsp sequences into a single string. RAxML (HPC-HYBRID-AVX2) was called with the PROTCATBLOSUM62 model, 1,000 bootstraps, and the -f a hill-climbing options enabled. Final trees were rendered using the ITOL software suite (Fig. S1).

### Assessment of sequence conservation using Shannon entropy.

To evaluate the conservation of residues in each Wsp protein, alignments were generated as described above for the Wsp phylogeny except that C. crescentus was not included in the alignments. The generated alignments were uploaded to the Protein Residue Conservation Prediction tool established by Capra and Singh to calculate the Shannon entropy of each position within the alignment (57). We used the Shannon entropy scoring method with a window size of 3, sequence weighting enabled, and BLOSUM62 matrix options. This tool evaluates the entropy in an alignment where sites with high variation have high Shannon entropy. The tool then scales the entropy to a range of (0,1) and subtracts this score from 1 so that higher scores indicate greater conservation. This value is referred to as the conservation score. The conservation scores provided by this analysis were then downloaded, interpreted via Python, and plotted via the matplotlib library for each Wsp alignment. Residues with a high conservative threshold (≥0.80) conservation score are highlighted in red. Available single-nucleotide polymorphism (SNP) data and annotation data were mapped to this graph to provide context on the function of these identified regions. We accomplished this by generating a consensus sequence for each alignment using the SeqIO library of BioPython v-1.78 (71) (code provided in File S1 at https://dsc.duq.edu/biology-data/4/). We then individually mapped all available mutation data in the literature to the consensus sequences on a case-by-case basis using MEGA alignment software (76) (Table S3). This provided a residue position of where mutations would fall in the alignment. Likewise, the consensus sequences were annotated using available Wsp literature (18, 27, 28, 32–34, 37, 39, 45–47) and the NCBI’s Conserved Domain Database (CDD) (77) and through homology to the enteric chemotaxis Che system as reported in Table S4.

### Identifying residues essential for divergent H-system signaling.

Alignments were constructed for the seven Wsp proteins (WspA, WspB, WspC, WspD, WspE, WspF, and WspH/R) using the 794 peptide sequences as detailed above. The alignment for each Wsp protein was parsed via Python scripts into an H- or R-system alignment subset, resulting in 206 or 588 peptides per alignment, respectively. The parsing method retained the organization of the original 794-sequence alignment, allowing the H- and R-system alignment subsets to be directly compared for each Wsp protein. We generated a consensus sequence for all 14 alignments (7 Wsp proteins parsed into H- or R-system subsets) using the consensus algorithm of the AlignIO library in BioPython (71). Next, we calculated the conservation scores of the alignments as described above. We used the generated consensus sequence and the conservation score to create residue pairs for each position in the alignment, creating 2,899 residue pairs. For example, residue 360 of WspA has a serine in the H-system (conservation score = 0.99999) but a lysine in the R-system (conservation score = 0.80182). We identified residues that had a conservation score of ≥0.80 in both the H- and R-system data sets, which indicates that the residue is likely functionally important in both systems. This reduced the data set to 971 residue pairs. We next considered mismatched residue pairs where a mutation likely occurred within the H-system during or after the evolutionary divergence of the H- and R-systems, further reducing the data set to 178 residue pairs. Fifty-six ambiguous residue pairs, or residues assigned “X” in the consensus sequence, were omitted from our analysis, resulting in 122 residue pairs. We used the data set of Pechmann and Frydman to identify conservative or nonconservative mutations in the H-system subset compared to the R-system subset (63). Seventy-three residue pairs constituted a conservative mutation from the R-system to the H-system and were filtered out of our data set. The final data set contained 43 residue pairs where each residue is highly conserved in its respective alignment subset; the residue pair identifies a nonconservative mutation within the H-system compared to the R-system that may be essential for H-system signaling.

### Motif assessment of WspB and WspD.

Assessment of conservation scores for WspB and WspD found that the conserved sites of these proteins were not similar. To visualize this difference, we collected the conservation scores of residues with a high conservative threshold (≥0.80) that were continuous for 3 or more residues. The identified islands of conservation plus 2 flanking residues on both ends were selected from the alignment to generate a sequence logo. Sequence logos were generated using WebLogo v-2.8.2 (78) with default settings and the logo range values for the sequences of interest. Comparisons of the motif sequence logos of WspB and WspD were conducted manually.

### Data availability.

All sequences assessed in this study were obtained from the NCBI’s Sequence Read Archive (SRA). The following files of this study are openly available in the Duquesne Scholarship Collection repository at https://dsc.duq.edu/biology-data/4/: Data Set S1 (MultiGeneBlast metadata for the P. fluorescens Pf0-1 *wsp* operon), Data Set S2 (MultiGeneBlast metadata for the B. cenocepacia HI2424 *wsp* gene cluster), File S1 (Python script executed on Data Sets S1 and S2), File S2 (R script executed by File S1 to download RefSeq genome assemblies), File S3 (R script executed by File S1 to parse the File S2 output for Bac120 set creation), and File S4 (R script executed by File S1 to parse the File S3 output and finalize the Bac120 data set).

## References

[1] Flemming H-C, Wingender J. 2010. The biofilm matrix. Nat Rev Microbiol 8:623–633. 10.1038/nrmicro2415. 20676145

[2] Sutherland I. 2001. The biofilm matrix—an immobilized but dynamic microbial environment. Trends Microbiol 9:222–227. 10.1016/s0966-842x(01)02012-1. 11336839

[3] Schlafer S, Meyer RL. 2017. Confocal microscopy imaging of the biofilm matrix. J Microbiol Methods 138:50–59. 10.1016/j.mimet.2016.03.002. 26979645

[4] Limoli DH, Jones CJ, Wozniak DJ. 2015. Bacterial extracellular polysaccharides in biofilm formation and function. Microbiol Spectr 3:MB-0011-2014. 10.1128/microbiolspec.MB-0011-2014. PMC4657554 26185074

[5] Fong JNC, Yildiz FH. 2015. Biofilm matrix proteins. Microbiol Spectr 3:MB-0004-2014. 10.1128/microbiolspec.MB-0004-2014. PMC4480581 26104709

[6] Hobley L, Harkins C, MacPhee CE, Stanley-Wall NR. 2015. Giving structure to the biofilm matrix: an overview of individual strategies and emerging common themes. FEMS Microbiol Rev 39:649–669. 10.1093/femsre/fuv015. 25907113 PMC4551309

[7] Branda SS, Vik Å, Friedman L, Kolter R. 2005. Biofilms: the matrix revisited. Trends Microbiol 13:20–26. 10.1016/j.tim.2004.11.006. 15639628

[8] Hou L, Debru A, Chen Q, Bao Q, Li K. 2019. AmrZ regulates swarming motility through cyclic di-GMP-dependent motility inhibition and controlling Pel polysaccharide production in *Pseudomonas aeruginosa* PA14. Front Microbiol 10:1847. 10.3389/fmicb.2019.01847. 31474950 PMC6707383

[9] Jayathilake PG, Jana S, Rushton S, Swailes D, Bridgens B, Curtis T, Chen J. 2017. Extracellular polymeric substance production and aggregated bacteria colonization influence the competition of microbes in biofilms. Front Microbiol 8:1865. 10.3389/fmicb.2017.01865. 29021783 PMC5623813

[10] Di Martino P. 2018. Extracellular polymeric substances, a key element in understanding biofilm phenotype. AIMS Microbiol 4:274–288. 10.3934/microbiol.2018.2.274. 31294215 PMC6604936

[11] Dieltjens L, Appermans K, Lissens M, Lories B, Kim W, Van der Eycken EV, Foster KR, Steenackers HP. 2020. Inhibiting bacterial cooperation is an evolutionarily robust anti-biofilm strategy. Nat Commun 11:107. 10.1038/s41467-019-13660-x. 31919364 PMC6952394

[12] Decho AW, Gutierrez T. 2017. Microbial extracellular polymeric substances (EPSs) in ocean systems. Front Microbiol 8:922. 10.3389/fmicb.2017.00922. 28603518 PMC5445292

[13] Tischler AD, Camilli A. 2004. Cyclic diguanylate (c-di-GMP) regulates *Vibrio cholerae* biofilm formation. Mol Microbiol 53:857–869. 10.1111/j.1365-2958.2004.04155.x. 15255898 PMC2790424

[14] Kirillina O, Fetherston JD, Bobrov AG, Abney J, Perry RD. 2004. HmsP, a putative phosphodiesterase, and HmsT, a putative diguanylate cyclase, control Hms-dependent biofilm formation in *Yersinia pestis*. Mol Microbiol 54:75–88. 10.1111/j.1365-2958.2004.04253.x. 15458406

[15] García B, Latasa C, Solano C, Portillo FG, Gamazo C, Lasa I. 2004. Role of the GGDEF protein family in *Salmonella* cellulose biosynthesis and biofilm formation. Mol Microbiol 54:264–277. 10.1111/j.1365-2958.2004.04269.x. 15458421

[16] Pesavento C, Hengge R. 2009. Bacterial nucleotide-based second messengers. Curr Opin Microbiol 12:170–176. 10.1016/j.mib.2009.01.007. 19318291

[17] Römling U, Galperin MY, Gomelsky M. 2013. Cyclic di-GMP: the first 25 years of a universal bacterial second messenger. Microbiol Mol Biol Rev 77:1–52. 10.1128/MMBR.00043-12. 23471616 PMC3591986

[18] O’Connor JR, Kuwada NJ, Huangyutitham V, Wiggins PA, Harwood CS. 2012. Surface sensing and lateral subcellular localization of WspA, the receptor in a chemosensory-like system leading to c-di-GMP production. Mol Microbiol 86:720–729. 10.1111/mmi.12013. 22957788 PMC3501340

[19] Jiménez-Fernández A, López-Sánchez A, Calero P, Govantes F. 2015. The c-di-GMP phosphodiesterase BifA regulates biofilm development in *Pseudomonas putida*. Environ Microbiol Rep 7:78–84. 10.1111/1758-2229.12153. 25870874

[20] Jenal U, Reinders A, Lori C. 2017. Cyclic di-GMP: second messenger extraordinaire. Nat Rev Microbiol 15:271–284. 10.1038/nrmicro.2016.190. 28163311

[21] Simm R, Morr M, Kader A, Nimtz M, Römling U. 2004. GGDEF and EAL domains inversely regulate cyclic di-GMP levels and transition from sessility to motility. Mol Microbiol 53:1123–1134. 10.1111/j.1365-2958.2004.04206.x. 15306016

[22] Malone J. 2015. Role of small colony variants in persistence of *Pseudomonas aeruginosa* infections in cystic fibrosis lungs. Infect Drug Resist 8:237–247. 10.2147/IDR.S68214. 26251621 PMC4524453

[23] Flynn KM, Dowell G, Johnson TM, Koestler BJ, Waters CM, Cooper VS. 2016. Evolution of ecological diversity in biofilms of *Pseudomonas aeruginosa* by altered cyclic diguanylate signaling. J Bacteriol 198:2608–2618. 10.1128/JB.00048-16. 27021563 PMC5019052

[24] Malone JG, Jaeger T, Manfredi P, Dötsch A, Blanka A, Bos R, Cornelis GR, Häussler S, Jenal U. 2012. The YfiBNR signal transduction mechanism reveals novel targets for the evolution of persistent *Pseudomonas aeruginosa* in cystic fibrosis airways. PLoS Pathog 8:e1002760. 10.1371/journal.ppat.1002760. 22719254 PMC3375315

[25] Malone JG, Jaeger T, Spangler C, Ritz D, Spang A, Arrieumerlou C, Kaever V, Landmann R, Jenal U. 2010. YfiBNR mediates cyclic di-GMP dependent small colony variant formation and persistence in *Pseudomonas aeruginosa*. PLoS Pathog 6:e1000804. 10.1371/journal.ppat.1000804. 20300602 PMC2837407

[26] Häussler S, Tümmler B, Weissbrodt H, Rohde M, Steinmetz I. 1999. Small-colony variants of *Pseudomonas aeruginosa* in cystic fibrosis. Clin Infect Dis 29:621–625. 10.1086/598644. 10530458

[27] Hickman JW, Tifrea DF, Harwood CS. 2005. A chemosensory system that regulates biofilm formation through modulation of cyclic diguanylate levels. Proc Natl Acad Sci USA 102:14422–14427. 10.1073/pnas.0507170102. 16186483 PMC1234902

[28] Bantinaki E, Kassen R, Knight CG, Robinson Z, Spiers AJ, Rainey PB. 2007. Adaptive divergence in experimental populations of *Pseudomonas fluorescens*. III. Mutational origins of wrinkly spreader diversity. Genetics 176:441–453. 10.1534/genetics.106.069906. 17339222 PMC1893022

[29] Irie Y, Borlee BR, O’Connor JR, Hill PJ, Harwood CS, Wozniak DJ, Parsek MR. 2012. Self-produced exopolysaccharide is a signal that stimulates biofilm formation in *Pseudomonas aeruginosa*. Proc Natl Acad Sci USA 109:20632–20636. 10.1073/pnas.1217993109. 23175784 PMC3528562

[30] Ha D-G, O’Toole GA. 2015. c-di-GMP and its effects on biofilm formation and dispersion: a *Pseudomonas aeruginosa* review. Microbiol Spectr 3:MB-0003-2014. 10.1128/microbiolspec.MB-0003-2014. PMC4498269 26104694

[31] Rybtke MT, Borlee BR, Murakami K, Irie Y, Hentzer M, Nielsen TE, Givskov M, Parsek MR, Tolker-Nielsen T. 2012. Fluorescence-based reporter for gauging cyclic di-GMP levels in *Pseudomonas aeruginosa*. Appl Environ Microbiol 78:5060–5069. 10.1128/AEM.00414-12. 22582064 PMC3416407

[32] Goymer P, Kahn SG, Malone JG, Gehrig SM, Spiers AJ, Rainey PB. 2006. Adaptive divergence in experimental populations of *Pseudomonas fluorescens*. II. Role of the GGDEF regulator WspR in evolution and development of the wrinkly spreader phenotype. Genetics 173:515–526. 10.1534/genetics.106.055863. 16624907 PMC1526540

[33] Spiers AJ, Kahn SG, Bohannon J, Travisano M, Rainey PB. 2002. Adaptive divergence in experimental populations of *Pseudomonas fluorescens*. I. Genetic and phenotypic bases of wrinkly spreader fitness. Genetics 161:33–46. 10.1093/genetics/161.1.33. 12019221 PMC1462107

[34] Güvener ZT, Harwood CS. 2007. Subcellular location characteristics of the *Pseudomonas aeruginosa* GGDEF protein, WspR, indicate that it produces cyclic-di-GMP in response to growth on surfaces. Mol Microbiol 66:1459–1473. 10.1111/j.1365-2958.2007.06008.x. 18028314 PMC4105145

[35] De N, Pirruccello M, Krasteva PV, Bae N, Raghavan RV, Sondermann H. 2008. Phosphorylation-independent regulation of the diguanylate cyclase WspR. PLoS Biol 6:e67. 10.1371/journal.pbio.0060067. 18366254 PMC2270323

[36] Newell PD, Yoshioka S, Hvorecny KL, Monds RD, O’Toole GA. 2011. Systematic analysis of diguanylate cyclases that promote biofilm formation by *Pseudomonas fluorescens* Pf0-1. J Bacteriol 193:4685–4698. 10.1128/JB.05483-11. 21764921 PMC3165641

[37] Huangyutitham V, Güvener ZT, Harwood CS. 2013. Subcellular clustering of the phosphorylated WspR response regulator protein stimulates its diguanylate cyclase activity. mBio 4:e00242-13. 10.1128/mBio.00242-13. 23653447 PMC3663191

[38] Armbruster CR, Lee CK, Parker-Gilham J, De Anda J, Xia A, Zhao K, Murakami K, Tseng BS, Hoffman LR, Jin F, Harwood CS, Wong GC, Parsek MR. 2019. Heterogeneity in surface sensing suggests a division of labor in *Pseudomonas aeruginosa* populations. Elife 8:e45084. 10.7554/eLife.45084. 31180327 PMC6615863

[39] Kim W, Levy SB, Foster KR. 2016. Rapid radiation in bacteria leads to a division of labour. Nat Commun 7:10508. 10.1038/ncomms10508. 26852925 PMC4748119

[40] Chang C-Y. 2017. Surface sensing for biofilm formation in *Pseudomonas aeruginosa*. Front Microbiol 8:2671. 10.3389/fmicb.2017.02671. 29375533 PMC5767216

[41] Malone JG, Williams R, Christen M, Jenal U, Spiers AJ, Rainey PB. 2007. The structure-function relationship of WspR, a *Pseudomonas fluorescens* response regulator with a GGDEF output domain. Microbiology (Reading) 153:980–994. 10.1099/mic.0.2006/002824-0. 17379708

[42] Francis VI, Stevenson EC, Porter SL. 2017. Two-component systems required for virulence in *Pseudomonas aeruginosa*. FEMS Microbiology Lett 364:fnx104. 10.1093/femsle/fnx104. PMC5812489 28510688

[43] Cooper VS, Staples RK, Traverse CC, Ellis CN. 2014. Parallel evolution of small colony variants in *Burkholderia cenocepacia* biofilms. Genomics 104:447–452. 10.1016/j.ygeno.2014.09.007. 25263109

[44] O’Rourke D, FitzGerald CE, Traverse CC, Cooper VS. 2015. There and back again: consequences of biofilm specialization under selection for dispersal. Front Genet 6:18. 10.3389/fgene.2015.00018. 25717335 PMC4324302

[45] Spiers AJ, Bohannon J, Gehrig SM, Rainey PB. 2003. Biofilm formation at the air-liquid interface by the *Pseudomonas fluorescens* SBW25 wrinkly spreader requires an acetylated form of cellulose. Mol Microbiol 50:15–27. 10.1046/j.1365-2958.2003.03670.x. 14507360

[46] McDonald MJ, Gehrig SM, Meintjes PL, Zhang X, Rainey PB. 2009. Adaptive divergence in experimental populations of *Pseudomonas fluorescens*. IV. Genetic constraints guide evolutionary trajectories in a parallel adaptive radiation. Genetics 183:1041–1053. 10.1534/genetics.109.107110. 19704015 PMC2778958

[47] Yan J, Deforet M, Boyle KE, Rahman R, Liang R, Okegbe C, Dietrich LEP, Qiu W, Xavier JB. 2017. Bow-tie signaling in c-di-GMP: machine learning in a simple biochemical network. PLoS Comput Biol 13:e1005677. 10.1371/journal.pcbi.1005677. 28767643 PMC5555705

[48] Silva IN, Ferreira AS, Becker JD, Zlosnik JEA, Speert DP, He J, Mil-Homens D, Moreira LM. 2011. Mucoid morphotype variation of *Burkholderia multivorans* during chronic cystic fibrosis lung infection is correlated with changes in metabolism, motility, biofilm formation and virulence. Microbiology (Reading) 157:3124–3137. 10.1099/mic.0.050989-0. 21835880

[49] Zlosnik JEA, Costa PS, Brant R, Mori PYB, Hird TJ, Fraenkel MC, Wilcox PG, Davidson AGF, Speert DP. 2011. Mucoid and nonmucoid *Burkholderia cepacia* complex bacteria in cystic fibrosis infections. Am J Respir Crit Care Med 183:67–72. 10.1164/rccm.201002-0203OC. 20709823

[50] De N, Navarro MVAS, Raghavan RV, Sondermann H. 2009. Determinants for the activation and autoinhibition of the diguanylate cyclase response regulator WspR. J Mol Biol 393:619–633. 10.1016/j.jmb.2009.08.030. 19695263 PMC2760619

[51] Ortega DR, Fleetwood AD, Krell T, Harwood CS, Jensen GJ, Zhulin IB. 2017. Assigning chemoreceptors to chemosensory pathways in *Pseudomonas aeruginosa*. Proc Natl Acad Sci USA 114:12809–12814. 10.1073/pnas.1708842114. 29133402 PMC5715753

[52] Wuichet K, Zhulin IB. 2010. Origins and diversification of a complex signal transduction system in prokaryotes. Sci Signal 3:ra50. 10.1126/scisignal.2000724. 20587806 PMC3401578

[53] Hamer R, Chen P-Y, Armitage JP, Reinert G, Deane CM. 2010. Deciphering chemotaxis pathways using cross species comparisons. BMC Syst Biol 4:3. 10.1186/1752-0509-4-3. 20064255 PMC2829493

[54] Sampedro I, Parales RE, Krell T, Hill JE. 2015. *Pseudomonas* chemotaxis. FEMS Microbiol Rev 39:17–46. 10.1111/1574-6976.12081. 25100612

[55] Ravenhall M, Škunca N, Lassalle F, Dessimoz C. 2015. Inferring horizontal gene transfer. PLoS Comput Biol 11:e1004095. 10.1371/journal.pcbi.1004095. 26020646 PMC4462595

[56] Planet PJ. 2006. Tree disagreement: measuring and testing incongruence in phylogenies. J Biomed Inform 39:86–102. 10.1016/j.jbi.2005.08.008. 16243006

[57] Capra JA, Singh M. 2007. Predicting functionally important residues from sequence conservation. Bioinformatics 23:1875–1882. 10.1093/bioinformatics/btm270. 17519246

[58] Zhulin IB. 2001. The superfamily of chemotaxis transducers: from physiology to genomics and back. Adv Microb Physiol 45:157–198. 10.1016/s0065-2911(01)45004-1. 11450109

[59] Alexander RP, Zhulin IB. 2007. Evolutionary genomics reveals conserved structural determinants of signaling and adaptation in microbial chemoreceptors. Proc Natl Acad Sci USA 104:2885–2890. 10.1073/pnas.0609359104. 17299051 PMC1797150

[60] Feng X, Lilly AA, Hazelbauer GL. 1999. Enhanced function conferred on low-abundance chemoreceptor Trg by a methyltransferase-docking site. J Bacteriol 181:3164–3171. 10.1128/JB.181.10.3164-3171.1999. 10322018 PMC93772

[61] Huang Z, Pan X, Xu N, Guo M. 2019. Bacterial chemotaxis coupling protein: structure, function and diversity. Microbiol Res 219:40–48. 10.1016/j.micres.2018.11.001. 30642465

[62] Alexander RP, Lowenthal AC, Harshey RM, Ottemann KM. 2010. CheV: CheW-like coupling proteins at the core of the chemotaxis signaling network. Trends Microbiol 18:494–503. 10.1016/j.tim.2010.07.004. 20832320 PMC2975053

[63] Pechmann S, Frydman J. 2014. Interplay between chaperones and protein disorder promotes the evolution of protein networks. PLoS Comput Biol 10:e1003674. 10.1371/journal.pcbi.1003674. 24968255 PMC4072544

[64] D’Argenio DA, Calfee MW, Rainey PB, Pesci EC. 2002. Autolysis and autoaggregation in *Pseudomonas aeruginosa* colony morphology mutants. J Bacteriol 184:6481–6489. 10.1128/JB.184.23.6481-6489.2002. 12426335 PMC135425

[65] Starkey M, Hickman JH, Ma L, Zhang N, De Long S, Hinz A, Palacios S, Manoil C, Kirisits MJ, Starner TD, Wozniak DJ, Harwood CS, Parsek MR. 2009. *Pseudomonas aeruginosa* rugose small-colony variants have adaptations that likely promote persistence in the cystic fibrosis lung. J Bacteriol 191:3492–3503. 10.1128/JB.00119-09. 19329647 PMC2681918

[66] Gloag ES, Marshall CW, Snyder D, Lewin GR, Harris JS, Santos-Lopez A, Chaney SB, Whiteley M, Cooper VS, Wozniak DJ. 2019. *Pseudomonas aeruginosa* interstrain dynamics and selection of hyperbiofilm mutants during a chronic infection. mBio 10:e01698-19. 10.1128/mBio.01698-19. 31409682 PMC6692513

[67] Moradali MF, Ghods S, Rehm BHA. 2017. *Pseudomonas aeruginosa* lifestyle: a paradigm for adaptation, survival, and persistence. Front Cell Infect Microbiol 7:39. 10.3389/fcimb.2017.00039. 28261568 PMC5310132

[68] Irie Y, Starkey M, Edwards AN, Wozniak DJ, Romeo T, Parsek MR. 2010. *Pseudomonas aeruginosa* biofilm matrix polysaccharide Psl is regulated transcriptionally by RpoS and post-transcriptionally by RsmA. Mol Microbiol 78:158–172. 10.1111/j.1365-2958.2010.07320.x. 20735777 PMC2984543

[69] Blanka A, Düvel J, Dötsch A, Klinkert B, Abraham W-R, Kaever V, Ritter C, Narberhaus F, Häussler S. 2015. Constitutive production of c-di-GMP is associated with mutations in a variant of *Pseudomonas aeruginosa* with altered membrane composition. Sci Signal 8:ra36. 10.1126/scisignal.2005943. 25872871

[70] Medema MH, Takano E, Breitling R. 2013. Detecting sequence homology at the gene cluster level with MultiGeneBlast. Mol Biol Evol 30:1218–1223. 10.1093/molbev/mst025. 23412913 PMC3670737

[71] Cock PJA, Antao T, Chang JT, Chapman BA, Cox CJ, Dalke A, Friedberg I, Hamelryck T, Kauff F, Wilczynski B, de Hoon MJL. 2009. Biopython: freely available Python tools for computational molecular biology and bioinformatics. Bioinformatics 25:1422–1423. 10.1093/bioinformatics/btp163. 19304878 PMC2682512

[72] Parks DH, Rinke C, Chuvochina M, Chaumeil P-A, Woodcroft BJ, Evans PN, Hugenholtz P, Tyson GW. 2017. Recovery of nearly 8,000 metagenome-assembled genomes substantially expands the tree of life. Nat Microbiol 2:1533–1542. 10.1038/s41564-017-0012-7. 28894102

[73] Johnson LS, Eddy SR, Portugaly E. 2010. Hidden Markov model speed heuristic and iterative HMM search procedure. BMC Bioinformatics 11:431. 10.1186/1471-2105-11-431. 20718988 PMC2931519

[74] Nakamura T, Yamada KD, Tomii K, Katoh K. 2018. Parallelization of MAFFT for large-scale multiple sequence alignments. Bioinformatics 34:2490–2492. 10.1093/bioinformatics/bty121. 29506019 PMC6041967

[75] Capella-Gutierrez S, Silla-Martinez JM, Gabaldon T. 2009. trimAl: a tool for automated alignment trimming in large-scale phylogenetic analyses. Bioinformatics 25:1972–1973. 10.1093/bioinformatics/btp348. 19505945 PMC2712344

[76] Kumar S, Stecher G, Li M, Knyaz C, Tamura K. 2018. MEGA X: molecular evolutionary genetics analysis across computing platforms. Mol Biol Evol 35:1547–1549. 10.1093/molbev/msy096. 29722887 PMC5967553

[77] Lu S, Wang J, Chitsaz F, Derbyshire MK, Geer RC, Gonzales NR, Gwadz M, Hurwitz DI, Marchler GH, Song JS, Thanki N, Yamashita RA, Yang M, Zhang D, Zheng C, Lanczycki CJ, Marchler-Bauer A. 2020. CDD/SPARCLE: the conserved domain database in 2020. Nucleic Acids Res 48:D265–D268. 10.1093/nar/gkz991. 31777944 PMC6943070

[78] Crooks GE, Hon G, Chandonia J-M, Brenner SE. 2004. WebLogo: a sequence logo generator. Genome Res 14:1188–1190. 10.1101/gr.849004. 15173120 PMC419797

[79] Kim W, Racimo F, Schluter J, Levy SB, Foster KR. 2014. Importance of positioning for microbial evolution. Proc Natl Acad Sci U S A 111:E1639–E1647. 10.1073/pnas.1323632111. PMC4000849 24715732

