# Supplementary material for: Evolutionary Divergence of the Wsp Signal Transduction Systems in Beta- and Gammaproteobacteria
Source: Appl Environ Microbiol. 2021 Oct 28;87(22):e01306-21. doi: 10.1128/AEM.01306-21 (PMC8552884; doi:10.1128/AEM.01306-21)
Supplement: Supplemental file 1 — Figures S1 to S3, Tables S1 to S4. Download AEM.01306-21-s0001.pdf, PDF file, 0.8 MB [file aem.01306-21-s0001.pdf]

Supplemental Material

Supplemental Tables and Figures

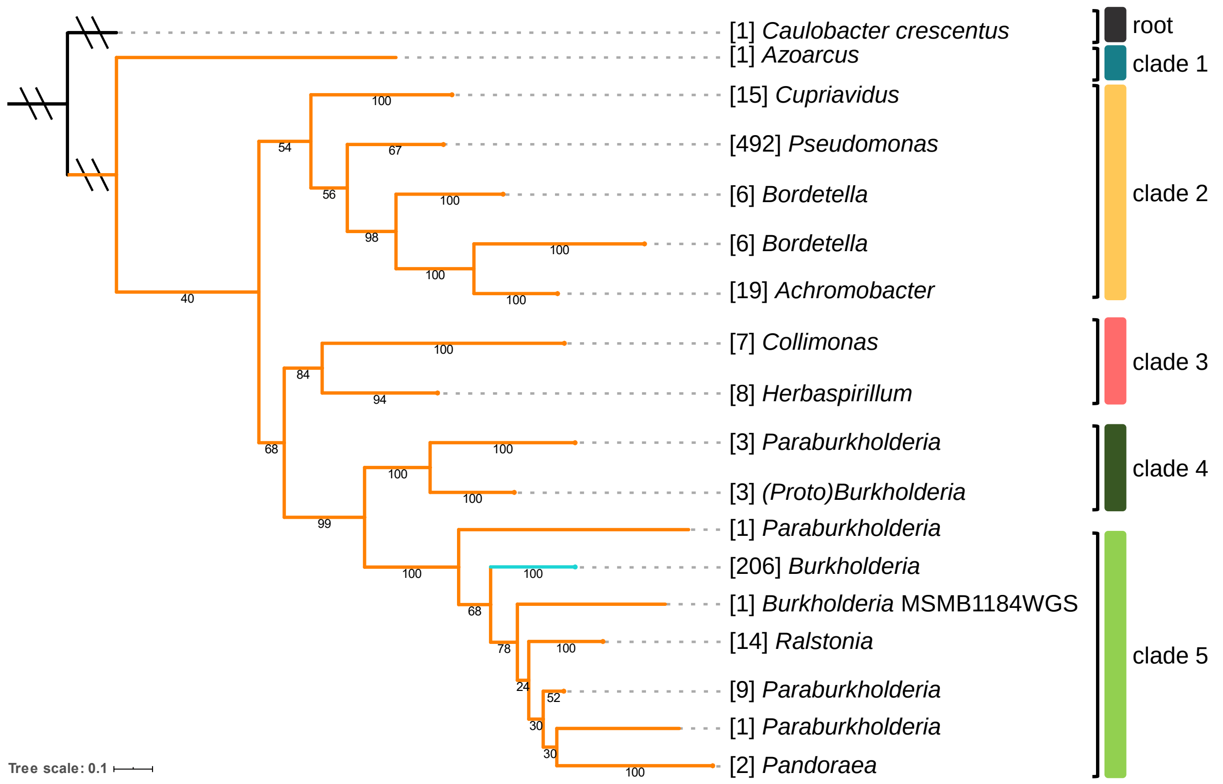

Figure S1. Phylogenetic analysis of the core Wsp proteins indicates that the R-system predates the H-system. The H-system (teal) and R-system (orange) gene tree was constructed using the amino acid sequences of the core Wsp proteins (WspA, WspB, WspC, WspD, WspE, and WspF). The phylogeny is rooted to Wsp homologs in *Caulobacter crescentus* reported in Table S2. The Wsp system diverges into five distinct clades. Each clade is assigned a unique color and the same color scheme is applied to Figs. 3 and 4 to denote the respective clades. The R-system precedes the H-system and all 206 H-systems form a single branch. The values within parentheses indicate the number of species/strains within each branch and those under each branch represent the bootstrap support values (out of 100).

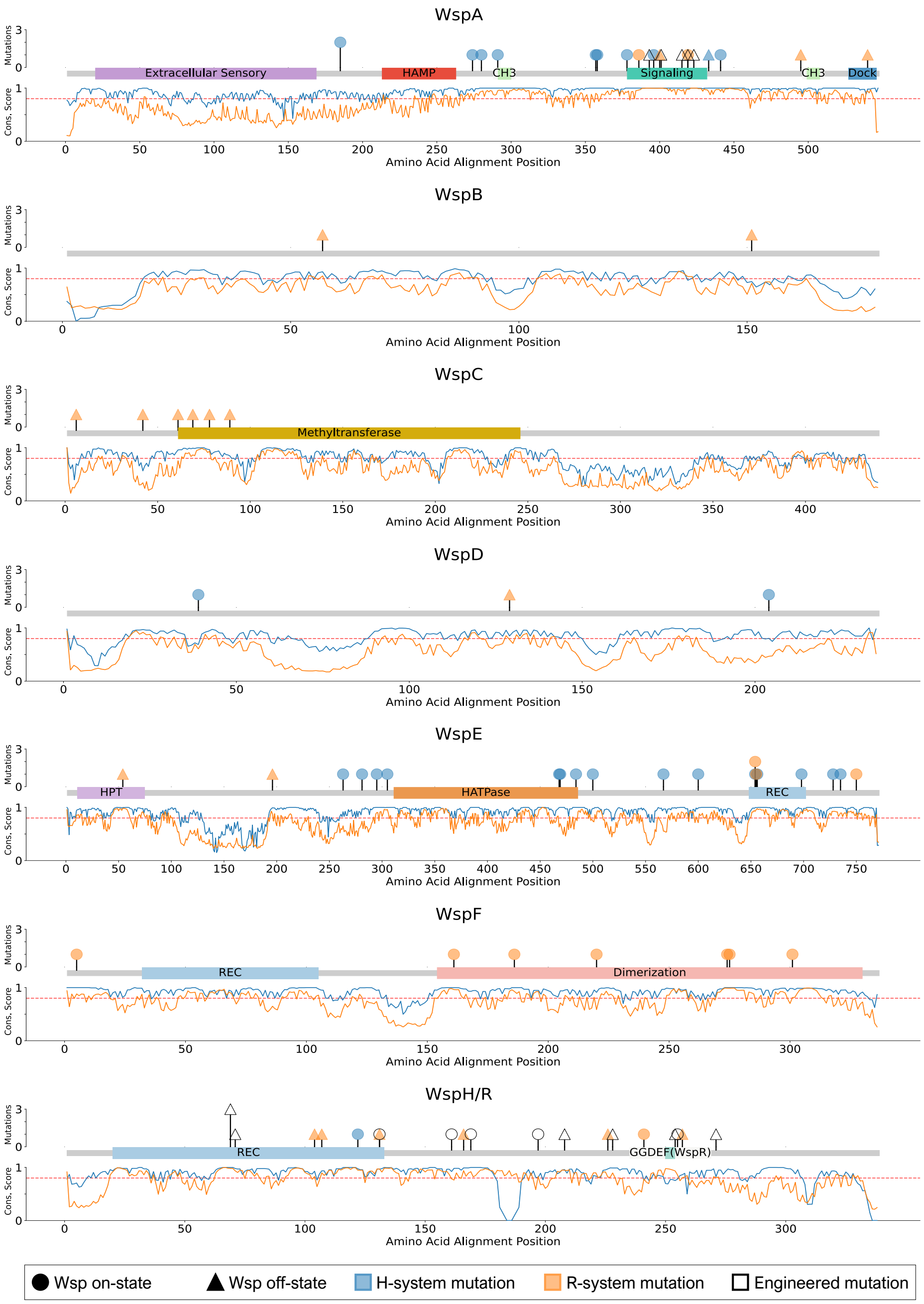

Figure S2. Discrete conservation of Wsp proteins within H- or R-systems. Amino acid sequences used to generate Figure 5 were divided into an H-system plot (blue) or an R-system plot (orange). Annotation data is derived from NCBI CDD (conserved domain database), Prosite, or Che homology as indicated in Table S4. Reported naturally occurring missense mutations from the literature in the H-system are shown in blue and those in the R-system are shown in orange. Engineered missense mutations reported in the literature are indicated in white. Mutations that turn on the respective Wsp system are indicated as circles while those that turn off the system are indicated as triangles. The y-axis represents the Shannon Entropy evaluation for each protein alignment where weighted values near 1 indicate high sequence conservation and values near zero indicate weak sequence conservation. The horizontal dashed line indicates where the weighted Shannon Entropy metric equals 0.8, denoting residues of greater functional or structural importance.

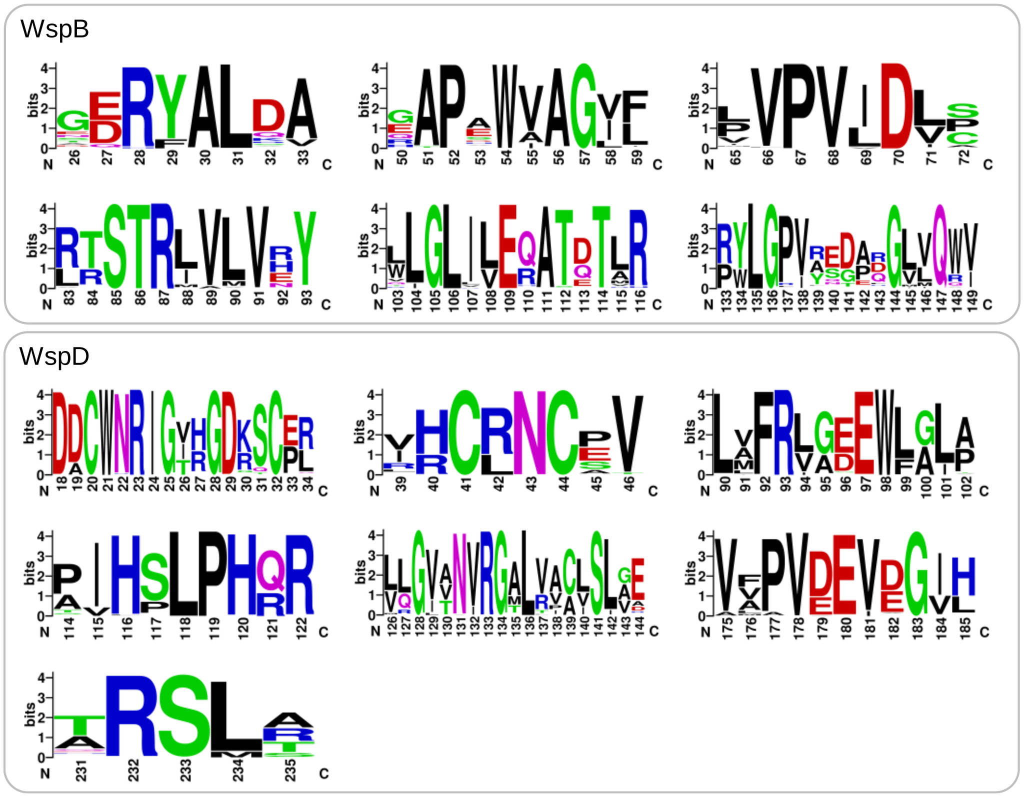

Figure S3. Sequence logos of conserved regions in WspB and WspD. Individual domains of high conservation between WspB and WspD show little to no similarity. Sequence logos were generated to represent the highly conserved regions in Figure 5 for WspB and WspD. Islands of high conservation were identified by the 0.8 Shannon entropy metric. Two residues immediately flanking the conserved region are included in the Sequence logos to ensure the entire conserved region is depicted. The values indicated on the x-axis denote the relative amino acid position within the coding sequence. No overlapping signatures were found between the WspB and WspD, which is surprising given their proposed similar function.

Table S1. Sequence conservation assessment of the Wsp signal transduction system and the enteric chemotaxis Che system.

				Average Conservation Score†	
Protein Identifier	*E. coli*	*P. fluorescens*	*B. cenocepacia*	Comparison of all 3 systems	Comparison of the H- and R-systems
Tsr/WspA	NP_415938	ABA72795	ABK10523	0.334	0.521
CheW/WspB	NP_416401	ABA72796	ABK10524	0.285	0.383
CheR/WspC	NP_416938	ABA72797	ABK10525	0.328	0.330
CheW/WspD	NP_416401	ABA72798	ABK10527	0.278	0.319
CheA/WspE	NP_416402	ABA72799	ABK10528	0.342	0.429
CheB/WspF	NP_416397	ABA72800	ABK10529	0.412	0.584
CheY/WspR/WspH	NP_416396	ABA72801	ABK10522	0.328	0.268

† Conservation scores assessed through Shannon entropy analyses (see Methods). Score ranges from 0 to 1 with 0 indicating no conservation and 1 indicating complete conservation. The score was determined for each residue in the alignment. The average conservation score of the protein alignment is reported for each system comparison.

Table S2. Establishing *C. crescentus* Wsp homologs as the root for the Wsp phylogenetic analysis.

C. crescentus	NCBI Accession	Homolog	Query †	Query Cover	E-value §	% Identity
CP001340.1	ACL96924	WspA	Pfl01_1052	60%	3.00E-29	29.31%
CP001340.1	ACL96585	WspB	Pfl01_1053	72%	4.00E-08	27.82%
CP001340.1	ACL93911	WspC	Pfl01_1054	44%	8.00E-25	32.66%
CP001340.1	ACL94859	WspD	Pfl01_1055	19%	1.00E-04	31.82%
CP001340.1	ACL94095	WspE	Pfl01_1056	78%	7.00E-68	30.36%
CP001340.1	ACL93912	WspF	Pfl01_1057	99%	1.00E-49	34.01%

† *P. fluorescens* Pf0-1 Wsp sequences were used as a query against *C. crescentus* CP001340.1 to identify respective homologs for rooting the Wsp phylogeny.
§ Identified proteins scored low E-values, indicating that they are acceptable homologs for rooting the Wsp phylogeny.

Table S3. Previously reported non-synonymous substitutions in Wsp systems and associated phenotypes.

Protein	Reference Information †					Assessment of Reference	
	System	Mutation	Type of Mutation	Organism	Reference	Wsp Signaling State §	Consensus Residue *
WspA	H-system	I196M	natural	*B. cenocepacia* HI2424	(1)	Active (high biofilm)	185
	H-system	I196N	natural	*B. cenocepacia* HI2424	(1)	Active (high biofilm)	185
	H-system	S285W	natural	*B. cenocepacia* HI2424	(1)	Active (high biofilm)	274
	H-system	S291*	natural	*B. cenocepacia* HI2424	(2)	Active (high biofilm)	280
	H-system	Q302H	natural	*B. cenocepacia* HI2424	(1)	Active (high biofilm)	291
	H-system	A368V	natural	*B. cenocepacia* HI2424	(1)	Active (high biofilm)	357
	H-system	A369V	natural	*B. cenocepacia* HI2424	(1)	Active (high biofilm)	358
	H-system	V389A	natural	*B. cenocepacia* HI2424	(1)	Active (high biofilm)	378
	R-system	A381V	natural	*P. fluorescens* Pf0-1	(3)	Active (wrinkled phenotype)	386
	R-system	S390A	engineered	*P. aeruginosa* PAO1	(4)	Inactive (assay)	393
	H-system	A407V	natural	*B. cenocepacia* HI2424	(1)	Active (high biofilm)	396
	R-system	A397V	natural	*P. aeruginosa* PA14	(5)	Inactive (swarming)	400
	R-system	E398A	engineered	*P. aeruginosa* PAO1	(4)	Inactive (assay)	401
	R-system	T412D	engineered	*P. aeruginosa* PAO1	(4)	Inactive (assay)	415
	R-system	R416N	engineered	*P. aeruginosa* PAO1	(4)	Inactive (assay)	419
	R-system	A418E	natural	*P. aeruginosa* PA14	(5)	Inactive (swarming)	421
	R-system	Q420R	engineered	*P. aeruginosa* PAO1	(4)	Inactive (assay)	423
	H-system	Q444*	natural	*B. cenocepacia* HI2424	(2)	Inactive (phenotype)	433
	H-system	A452V	natural	*B. cenocepacia* HI2424	(1)	Active (high biofilm)	441
	R-system	A506P	natural	*B. cenocepacia* HI2424	(2)	Inactive (phenotype)	495
	R-system	V537L	natural	*P. aeruginosa* PA14	(5)	Inactive (swarming)	540
WspB	R-system	V53G	natural	*P. aeruginosa* PA14	(5)	Inactive (swarming)	57
	R-system	V147G	natural	*P. aeruginosa* PA14	(5)	Inactive (swarming)	151
WspC	R-system	N2D	natural	*P. aeruginosa* PA14	(5)	Inactive (swarming)	6
	R-system	L38R	natural	*P. aeruginosa* PA14	(5)	Inactive (swarming)	42
	R-system	E54D	natural	*P. aeruginosa* PA14	(5)	Inactive (swarming)	61
	R-system	V62M	natural	*P. aeruginosa* PA14	(5)	Inactive (swarming)	69
	R-system	R71C	natural	*P. aeruginosa* PA14	(5)	Inactive (swarming)	78
	R-system	L82P	natural	*P. aeruginosa* PA14	(5)	Inactive (swarming)	89
WspD	H-system	L35P	natural	*B. cenocepacia* HI2424	(1)	Active (high biofilm)	39
	R-system	V123G	natural	*P. aeruginosa* PA14	(5)	Inactive (swarming)	129
	H-system	A202P	natural	*B. cenocepacia* HI2424	(1)	Active (high biofilm)	204
WspE	R-system	K55N	natural	*B. cenocepacia* HI2424	(2)	Inactive (phenotype)	54
	R-system	V194G	natural	*P. aeruginosa* PA14	(5)	Inactive (swarming)	196
	H-system	R261W	natural	*B. cenocepacia* HI2424	(1)	Active (high biofilm)	263
	H-system	D279V	natural	*B. cenocepacia* HI2424	(1)	Active (high biofilm)	281
	H-system	Y293H	natural	*B. cenocepacia* HI2424	(1)	Active (high biofilm)	295
	H-system	P303L	natural	*B. cenocepacia* HI2424	(1)	Active (high biofilm)	305
	H-system	A466T	natural	*B. cenocepacia* HI2424	(1)	Active (high biofilm)	468
	H-system	V467L	natural	*B. cenocepacia* HI2424	(1)	Active (high biofilm)	469
	H-system	A498V	natural	*B. cenocepacia* HI2424	(1)	Active (high biofilm)	500
	H-system	E565D	natural	*B. cenocepacia* HI2424	(1)	Active (high biofilm)	567
	H-system	A598S	natural	*B. cenocepacia* HI2424	(1)	Active (high biofilm)	600
	H-system	D652N	natural	*B. cenocepacia* HI2424	(1)	Active (high biofilm)	654
	R-system	D638G	natural	*P. fluorescens* SBW25	(6)	Active (Phenotype)	654
	R-system	D638Y	natural	*P. fluorescens* SBW25	(6)	Active (Phenotype)	654
	R-system	D648G	natural	*P. fluorescens* Pf0-1	(3)	Active (Phenotype)	655
	H-system	S654L	natural	*B. cenocepacia* HI2424	(1)	Active (high biofilm)	656
	H-system	D696G	natural	*B. cenocepacia* HI2424	(1)	Active (high biofilm)	698
	H-system	S726L	natural	*B. cenocepacia* HI2424	(7)	Active (high biofilm)	728
	H-system	D733V	natural	*B. cenocepacia* HI2424	(1)	Active (high biofilm)	735
	R-system	K734N	natural	*P. fluorescens* SBW25	(6)	Active (wrinkled phenotype)	750
WspF	R-system	I5S	natural	*P. fluorescens* SBW25	(8)	Active (wrinkled phenotype)	5
	R-system	G161D	natural	*P. fluorescens* SBW25	(8)	Active (wrinkled phenotype)	161
	R-system	H186Y	natural	*P. fluorescens* SBW25	(8)	Active (wrinkled phenotype)	186
	R-system	V220L	natural	*P. fluorescens* SBW25	(8)	Active (wrinkled phenotype)	220
	R-system	T274I	natural	*P. fluorescens* SBW25	(8)	Active (wrinkled phenotype)	274
	R-system	G275C	natural	*P. fluorescens* SBW25	(8)	Active (wrinkled phenotype)	275
	R-system	S301R	natural	*P. fluorescens* SBW25	(8)	Active (wrinkled phenotype)	301
WspH/R	H-system	L135F	natural	*B. cenocepacia* HI2424	(1)	Active (high biofilm)	122
	R-system	D70N	engineered	*P. aeruginosa* PAO1	(9)	Inactive (phenotype)	69
	R-system	D70A	engineered	*P. aeruginosa* PAO1	(10)	Inactive (assay)	69
	R-system	D67N	engineered	*P. fluorescens* SBW25	(11)	Inactive (phenotype)	69
	R-system	V72D	engineered	*P. aeruginosa* PAO1	(10)	Inactive (assay)	71
	R-system	P105L	natural	*P. aeruginosa* PA14	(5)	Inactive (swarming)	104
	R-system	K108E	natural	*P. aeruginosa* PA14	(5)	Inactive (swarming)	107
	R-system	R132W	natural	*P. aeruginosa* PA14	(5)	Inactive (swarming)	131
	R-system	R129C	engineered	*P. fluorescens* SBW25	(11)	Active (wrinkled phenotype)	131
	R-system	D159G	engineered	*P. fluorescens* SBW25	(11)	Active (wrinkled phenotype)	161
	R-system	L167D	natural	*P. aeruginosa* PAO1	(10)	Inactive (assay)	166
	R-system	L170D	engineered	*P. aeruginosa* PAO1	(10)	Active (assay)	169
	R-system	R198A	engineered	*P. aeruginosa* PAO1	(10)	Active (assay)	197
	R-system	D206G	engineered	*P. fluorescens* SBW25	(11)	Inactive (phenotype)	208
	R-system	E227V	natural	*P. aeruginosa* PA14	(5)	Inactive (swarming)	226
	R-system	L226S	engineered	*P. fluorescens* SBW25	(11)	Inactive (swarming)	228
	R-system	R242A	natural	*P. aeruginosa* PAO1	(12)	Active (assay)	241
	R-system	F252S	engineered	*P. fluorescens* SBW25	(11)	Inactive (phenotype)	254
	R-system	E253A	engineered	*P. aeruginosa* PAO1	(10)	Active (assay)	255
	R-system	V258L	natural	*P. aeruginosa* PA14	(5)	Inactive (swarming)	257
	R-system	G269R	engineered	*P. fluorescens* SBW25	(11)	Active (wrinkled phenotype)	271

† An extensive literature review identified 80 *wsp* non-synonymous substitutions. We report the organism and the Wsp system (H/R) studied, reported mutation, and whether the mutations emerged naturally or were engineered.
§ References were assessed for an active/inactive state of Wsp signaling based on the interpretations of the respective authors and data presented (phenotypes, biochemical assays, biofilm assays).
* Amino acid sequences of each mutated protein were aligned to the consensus sequence of the 794 Wsp dataset to determine the respective residue position.

Table S4. Annotation of functional domains within the consensus sequence of the Wsp signaling system.

Protein	Predicted Domain §	Reference	CDD		BlastP				Prosite		
			Region †	E-value	Region †	E-value	Query ID	% Similarity (Positives)	Region †	Score	Query ID
WspA	Extracellular Sensory								20-169	7.695	PS50003
	HAMP		213-263	4.71E-03							
	+CH_3 Tar-2 site-1	(13)			291-300	8.00E-03	*n/a*	70.0%			
	Wsp Signaling Domain	(4)			387-423	2.00E-22	*n/a*	97.3%			
	Tsr Signaling Domain	(14)			378-432	6.00E-17	P02942	74.5%			
	+CH_3 Trg site-1	(13)			499-508	8.30E-01	*n/a*	50.0%			
WspC	Methyltransferase		61-246	1.30E-17							
WspE	HPT		11-75	5.59E-04							
	HATPase		315-486	6.96E-44							
	REC		648-702	2.04E-08							
WspF	REC		32-105	1.92E-05							
	Methylesterase		154-330	2.47E-43							
WspH/R	REC		20-133	1.74E-11							
	Enzymatic/GGDEF		173-332	3.80E-28							

§ Functional domains were predicted based on the assessment of previously reported studies of the Wsp system, bioinformatic prediction tools (NCBI CDD, conserved domain database), and functionally resolved homologous domains of the enteric chemotaxis (Che) system.

† Refers to the amino acid positions within the consensus sequence of the 794 Wsp peptide alignment.

Supplemental References

1. Cooper VS, Staples RK, Traverse CC, Ellis CN. 2014. Parallel evolution of small colony variants in *Burkholderia cenocepacia* biofilms. Genomics 104:447–452.
2. O'Rourke D, FitzGerald CE, Traverse CC, Cooper VS. 2015. There and back again: consequences of biofilm specialization under selection for dispersal. Frontiers in Genetics 6:1–14.
3. Kim W, Levy SB, Foster KR. 2016. Rapid radiation in bacteria leads to a division of labour. Nature Communications 7:1–10.
4. O'Connor JR, Kuwada NJ, Huangyutitham V, Wiggins PA, Harwood CS. 2012. Surface sensing and lateral subcellular localization of WspA, the receptor in a chemosensory-like system leading to c-di-GMP production. Molecular microbiology 86:720–729.
5. Yan J, Deforet M, Boyle KE, Rahman R, Liang R, Okegbe C, Dietrich LEP, Qiu W, Xavier JB. 2017. Bow-tie signaling in c-di-GMP: Machine learning in a simple biochemical network. PLOS Computational Biology 13:e1005677.
6. McDonald MJ, Gehrig SM, Meintjes PL, Zhang X-X, Rainey PB. 2009. Adaptive divergence in experimental populations of *Pseudomonas fluorescens*. IV. Genetic constraints guide evolutionary trajectories in a parallel adaptive radiation. Genetics 183:1041–1053.
7. Traverse CC, Mayo-Smith LM, Poltak SR, Cooper VS. 2013. Tangled bank of experimentally evolved *Burkholderia* biofilms reflects selection during chronic infections. Proceedings of the National Academy of Sciences 110:E250-E259
8. Bantinaki E, Kassen R, Knight CG, Robinson Z, Spiers AJ, Rainey PB. 2007. Adaptive divergence in experimental populations of *Pseudomonas fluorescens*. III. Mutational origins of wrinkly spreader diversity. Genetics 176:441–453.
9. Güvener ZT, Harwood CS. 2007. Subcellular location characteristics of the *Pseudomonas aeruginosa* GGDEF protein, WspR, indicate that it produces cyclic-di-GMP in response to growth on surfaces. Molecular microbiology 66:1459–1473.
10. Huangyutitham V, Güvener ZT, Harwood CS. 2013. Subcellular clustering of the phosphorylated WspR response regulator protein stimulates its diguanylate cyclase activity. mBio 4:1–8.
11. Goymer P, Kahn SG, Malone JG, Gehrig SM, Spiers AJ, Rainey PB. 2006. Adaptive divergence in experimental populations of *Pseudomonas fluorescens*. II. Role of the GGDEF regulator WspR in evolution and development of the wrinkly spreader phenotype. Genetics 173:515–526.
12. De N, Pirruccello M, Krasteva PV, Bae N, Raghavan RV, Sondermann H. 2008. Phosphorylation-independent regulation of the diguanylate cyclase WspR. PLoS Biology 6:e67.

12. Rice M, Dahlquist F. 1991.Sites of deamidation and methylation in Tsr, a bacterial chemotaxis sensory transducer. Journal of Biological Chemistry 266:9746-9753

14. Alexander RP, Zhulin IB. 2007. Evolutionary genomics reveals conserved structural determinants of signaling and adaptation in microbial chemoreceptors. Proceedings of the National Academy of Sciences 104:2885–2890.
